# New Perspective on Impact of Folic Acid Supplementation during Pregnancy on Neurodevelopment/Autism in the Offspring Children – A Systematic Review

**DOI:** 10.1371/journal.pone.0165626

**Published:** 2016-11-22

**Authors:** Yunfei Gao, Chao Sheng, Ri-hua Xie, Wen Sun, Elizabeth Asztalos, Diane Moddemann, Lonnie Zwaigenbaum, Mark Walker, Shi Wu Wen

**Affiliations:** 1 Department of Obstetrics and Gynecology, Nanfang Hospital of Southern Medical University, Guangzhou, Guangdong, China; 2 OMNI Research Group, Department of Obstetrics and Gynecology, University of Ottawa Faculty of Medicine, Ottawa, Canada; 3 Clinical Epidemiology Program, Ottawa Hospital Research Institute, Ottawa, Canada; 4 Hunan University of Medicine Department of Nursing, Huaihua, Hunan, China; 5 McLaughlin Center for Population Risk Assessment, University of Ottawa Faculty of Medicine, Ottawa, Canada; 6 Department of Obstetrics and Gynecology, Third Affiliated Hospital, Guangzhou Medical University, Guangzhou, China; 7 Centre for Mother, Infant and Child Research, Sunnybrook Health Sciences Centre, Toronto, Ontario, Canada; 8 Departments of Pediatrics and Obstetrics and Gynecology Faculty of Medicine, University of Toronto, Toronto, Canada; 9 Department of Pediatrics and Child Health and Neonatal Follow-up Program, University of Manitoba, Winnipeg, Canada; 10 Departments of Pediatrics and of Psychiatry, University of Alberta, Edmonton, Canada; 11 Autism Research Centre, Glenrose Rehabilitation Hospital, Edmonton, Canada; 12 School of Epidemiology, Public Health, and Preventive Medicine, University of Ottawa, Ottawa, Canada; University of Missouri Columbia, UNITED STATES

## Abstract

It has been conclusively established that folic acid supplementation prior to and during early pregnancy (up to 12 weeks of gestation) can prevent neural tube defects (NTDs). We hypothesized that folate effects may extend from neuro-structural defects to alterations in neuro-behavioural and emotional skills including autism spectrum disorders (ASDs) and other developmental disorders. The objective of this review was to comprehensively evaluate evidence on the impact of folic acid on neurodevelopment other than NTDs. We conducted an online search of relevant literature compiled by the National Library of Medicine from Medline and EMBASE (searched on Dec 31, 2014: http://www.ncbi.nlm.nih.gov/entrez/query/fcgi and http://www.elsevier.com/online-tools/embase). We first created 3 files (search restricted to English literature) using the following key words: 1) folate or folic acid (171322 papers identified by this search); 2) maternal or pregnancy or pregnant or gestation or gestational or prenatal or antenatal or periconception or periconceptional (1349219 papers identified by this search); and 3) autism or autism spectrum disorders or developmental delay or development or neurodevelopment or mental or cognitive or language or personal-social or gross motor or fine motor or behaviour or intellectual or intelligence or Bayley Scale (8268145 papers identified by this search). We then merged the 3 files and reviewed the papers that addressed these three issues simultaneously. A total of 22 original papers that examined the association between folic acid supplementation in human pregnancy and neurodevelopment/autism were identified after the screening, with 15 studies showing a beneficial effect of folic acid supplementation on neurodevelopment/autism, 6 studies showed no statistically significant difference, while one study showed a harmful effect in > 5 mg folic acid supplementation/day during pregnancy. Folic acid supplementation in pregnancy may have beneficial effects on the neurodevelopment of children beyond its proven effect on NTDs.

## Introduction

There are major emotional, societal, and economic implications of impaired neurodevelopment and/or autism in children [1, 2]. These children will often require specialized schooling and other community resources. Although the survival/life-span of these infants may not be seriously affected, many of them may need treatments throughout their lifetime, and the cost to the public health care system could be huge. When they reach adulthood, productivity is often lower than those with normal development, indirectly increasing the societal burden.

It has been established that supplementation with folic acid around the time of conception reduces the risk of neural tube defects (NTDs) in the offspring [3, 4, 5, 6]. However, whether folic acid has a similar effect on impaired neurodevelopment and/or autism remains elusive. This article therefore focuses on assessing the role of folic acid supplementation during pregnancy and folate metabolism on neurodevelopmental outcomes including autism spectrum disorders (ASDs), other than NTDs.

## Materials and Methods

### Search strategy

We conducted an online search of relevant literature compiled by the National Library of Medicine from Medline and EMBASE (searched on December 31, 2014 of the site: http://www.ncbi.nlm.nih.gov/entrez/query/fcgi and http://www.elsevier.com/online-tools/embase), with restriction to human studies. We first created 3 files (restricting our search to English literature) using the following key words: 1) folate or folic acid (171322 papers identified by this search); 2) maternal or pregnancy or pregnant or gestation or gestational or prenatal or antenatal or periconception or periconceptional (1349219 papers identified by this search); and 3) autism or autism spectrum disorders or developmental delay or development or neurodevelopment or mental or cognitive or language or personal-social or gross motor or fine motor or behaviour or intellectual or intelligence or Bayley Scale (8268145 papers identified by this search). We then merged the 3 files. All abstracts of the papers identified by merging the 3 files were screened by two independent reviewers in our group to exclude irrelevant studies (such as those on NTDs); because the causation between folic acid supplementation in pregnancy and NTDs has been established, our review is interested in outcomes other than NTDs.

### Study selection

We included randomized controlled trials (RCTs), cohort studies, and case control studies that examined the association between folic acid supplementation during pregnancy and neurodevelopment/autism in the offspring children. Data extraction was conducted independently and screened all records at the title level by two reviewers (Chao Sheng and Ri-hua Xie). To enhance sensitivity, records were only removed if both reviewers excluded at the title level. The second level of review was at the abstract level followed by another round of review at the full-text level. Two independent reviewers abstracted data using a standardized form. When there was a disagreement it was resolved by discussion with a third reviewer (Yunfei Gao). Corresponding authors were contacted via e-mail at least three times to obtain data if the outcome of the neurodevelopment/autism in the offspring children could not be readily abstracted from the publication.

### Study quality assessment

The quality of included cohort and case control studies was assessed with the Newcastle Ottawa Scale [7]. Using this checklist, Yunfei Gao evaluated each of the included articles, with additional inputs from Chao Sheng and Ri-hua Xie. The details are shown in Table 1. Divergent views were resolved by consulting a third reviewer.

**Table 1:** Quality assessment of cohort studies.

	Selection				Comparability		Outcome			Score
	1	2	3	4	1_a_	1_b_	1_c_	2_d_	3_e_	
**Tamura, 2005**[44]	√	√	√	√	√	-	√	√	-	7
**Wehby, 2008**[45]	√	√	√	√	√	-	√	√	-	7
**Del Río Garcia, 2009**[46]	√	√	√	√	√	-	√	-	-	6
**Julvez, 2009**[17]	√	√	√	√	√	-	√	√	-	7
**Valera-Gran D, 2014**[18]	√	√	√	√	√	√	√	√	√	9
**Glaser, 2010**[47]	√	√	√	√	√	√	√	√	√	9
**Roza, 2010**[16]	√	√	√	√	√	-	√	√	-	7
**Schlotz, 2010**[48]	√	√	√	√	√	-	√	√	-	7
**Veena, 2010**[49]	√	√	√	√	√	-	√	√	-	7
**Campoy, 2011**[50]	√	√	√	√	√	√	√	√	√	9
**Roth, 2011**[14]	√	√	√	√	√	-	√	√	√	8
**Chatzi, 2012**[13]	√	√	√	√	√	-	√	√	√	8
**Forns, 2012**[12]	√	-	√	√	-	√	√	√	√	7
**Steenweg-de, 2012**[15]	√	√	√	√	√	-	√	√	√	8
**Villamor, 2012**[11]	√	√	√	√	-	√	√	√	√	8
**Wu, 2012**[51]	√	√	√	√	√	√	√	√	√	9
**Surén, 2013**[8]	√	√	√	√	√	√	√	√	√	9
**Steenweg-de Graaff J, 2014**[52]	√	√	√	√	√	√	√	√	√	9

1. Representativeness of the exposed cohort; 2. Selection of the non-exposed cohort; 3. Ascertainment of exposure; 4. Demonstration that outcome of interest was not present at start of study

1a. study controls for age

1b. study controls for additional factors (maternal age; education level; maternal smokingsocial class; parity; child sex)

1c. Assessment of outcome

2d. Follow-up long enough for outcomes to occur

3e. Adequacy of follow up of cohorts (one star if follow-up>90%)

√ means meet this condition and 1 score

### Data extraction and synthesis

Data extracted from each study included the first author’s last name, publication year, main outcome, sample size, study design, age of children, effect of folic acid, and comments on the study. Because of major heterogeneity in original studies in terms of study design and outcome and exposure measurements, no attempt to summarize the effect by meta-analysis was made. When studies demonstrated conflicting findings on different outcome measures, the study was defined as “no association”.

## Results

### Literature search

**Fig 1:**
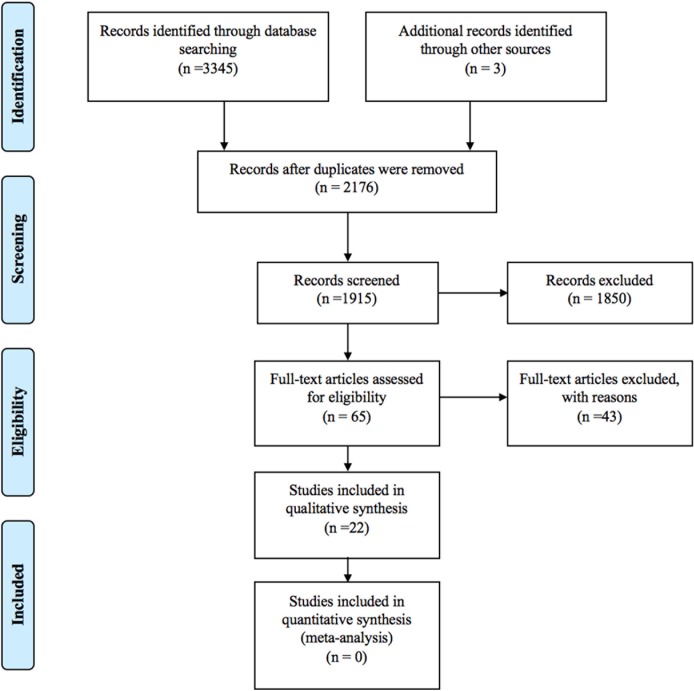
Records selection process.

A total of 3,348 papers were identified. Sixty five full-text articles were assessed for eligibility after screening. Most frequently the removed papers were animal studies or reviews or commentary/discussion in the interpretation of the study findings on other pregnancy outcomes (e.g., NTDs) or studies in humans but the effects of folic acid supplementation during pregnancy on neurodevelopment/autism in the offspring children was not examined. See details of selection in Fig 1.

A total of 22 original papers that looked at the association between folic acid supplementation in pregnancy and neurodevelopment/autism were identified after the screening in Table 2. Because of major heterogeneity in study design, exposure measurement, and outcome measurement, no attempt was made for quantitative synthesis of effect by meta-analysis. The 43 full-text excluded articles with reasons of exclusion were listed as supplement information in S1 Appendix.

**Table 2:** Summary of studies of the effects of folic acid supplementation in pregnancy on neurodevelopment and autism in offspring*.

Reference	Population	Exposure measurements	Results	Strength and Limitations
**Dobó 1998** [53]	RCT, 625 children followed to age of 2–6 years.	Preconceptional multivitamins containing 0.8 mg folic acid daily or placebo for at least 1 month before the planned conception and until the date of the second missed menstrual period.	No association. No differences in mental development between the two study groups.	Strengths: randomized, double-blind, placebo-controlled trial with long-term follow up.
**Christian, 2010** [43]	RCT, 676 children followed to age of 7 to 9 years.	Pregnant women were randomly allocatedto receive daily iron/folic acid (400 ug), iron/folic acid/zinc, or multiple micronutrients containing these plus 11 other micronutrients, or placebo (vitamin A only) groups from early pregnancy to 3 months postpartum.	Beneficial. The mean UNIT T score in the iron/folic acid group was 51.7 (SD, 8.5) and in the control group was 48.2 (SD, 10.2), with an adjusted mean difference of 2.38 (95% confidence interval [CI], 0.06–4.70; P = .04). In tests of executive function, scores were better in the iron/folic acid group relative to the control group for the Stroop test (adjusted mean difference in proportion who failed, −0.14; 95% CI, −0.23 to −0.04) and backward digit span (adjusted mean difference, 0.36; 95% CI, 0.01–0.71) but not for the go/no-go test. The MABC score was lower (better) in the iron/folic acid group compared with the control group but not after adjustment for confounders (mean difference, −1.47; 95% CI, −3.06 to 0.12; P = .07). Fingertapping test scores were higher (mean difference, 2.05; 95% CI, 0.87–3.24; P = .001) in the iron/folic acid group.	**Strengths**: 1. RCT with high rate of follow-up; 2. tests of general intelligence and motor function that are standardized for use in different cultures and settings.
**Tamura, 2005** [44]	Cohort study, 355 children followed to age of 5.3 years.	Supplementation of multivitamin/mineral tablet that contained 400 ug folic acid from 19 weeks of gestation until delivery and maternal blood folate concentrations.	No association. Folate status in late gestation assessed by plasma and erythrocyte folate had no impact on neurodevelopment of their children at age of 5.	Strengths: Measured both supplementation and maternal blood folate concentration. Limitations: 1. Small sample size; 2. low socioeconomic status, which may affect the generalizability of the study findings
**Wehby, 2008** [45]	Cohort study, 6,774 children followed to age of 3 years.	Multivitamins containing folic acid for at least 3 days a week during the 3 months prior to becoming aware of pregnancy and during the following 3 months.	No association. Folic acid use was associated with reduced odds for risk on the gross motor scale (OR = 0.5) but was also associated with a marginally significant increased odds for moderate risk on the personal-social scale (OR = 1.8).	**Strengths**: Large size from a national representative sample. **Limitations**: 1. No dosage information; 2. < 4% reported use of supplements; 3. no information on folic acid use alone; 4. screen tools instead of diagnostic tools.
**Del Río Garcia, 2009** [46]	Cohort study, 253 children followed to age of 1 year.	Folic acid food intake by food frequency questionnaire.	Beneficial. Deficiency in dietary folate intake was associated with a significant reduction of approximately 2 points in the mental development index in those whose mothers were carriers of the *TT* genotype (β = –1.8; 95% CI –3.6 to –0.04).	**Strengths: 1. Examined gene-environment interaction on** neurodevelopment. 2. Comparison of folate consumption between subjects who remained in the analysis and those who were lost or excluded did not show statistically significant differences. **Limitations:** 1. Potential selection bias as a function of the MTHFR 677C>T polymorphism; 2. Potential measurement error in the evaluation of dietary intake of folate.
**Julvez, 2009** [17]	Cohort study, 420 children followed to age of 4 years.	Folic acid supplement through structured interview at the end of the first trimester of pregnancy.	Beneficial. Folic acid supplementation during pregnancy was associated with improved verbal (b = 3.98, SE = 1.69), motor (b = 4.54, SE = 1.66) and verbal-executive function (b = 3.97, SE = 1.68) scores, social competence (b = 3.97, SE = 1.61), and inattention symptom [OR = 0.46; 95% CI 0.22, 0.95] scores.	**Strengths**: 1. Very high participation (94%) and (87%) follow-up rates; 2. Validated measurement scales. **Limitations**: 1. Residual confounding by unmeasured parental psychosocial variables; 2. Self-reported folic acid supplement use; 3. Lack of information on timing and dosage of folic acid supplement.
**Glaser, 2010** [47]	Cohort study, 5,344 children followed to age of 12 years.	Folic acid from supplementation and diet was assessed by questionnaires at 18 to 32 weeks of gestation.	No association. Maternal folic acid supplementation increased the odds of psychosis-like symptoms in children (odds ratio(OR) = 1.34; 95%-CI:[1.00;1.76]), children born to mothers with MTHFR C667T TT homozygous had decreased odds of psychosis-like symptoms (OR = 0.72; 95%CI:[0.50;1.02]; recessive model) with strongest effects in boys (OR = 0.44, 95%-CI:[0.22;0.79]; sex-specific p = 0.029). None of these associations remained significant after adjusting for multiple testing, however.	**Strengths**: 1. Large sample size; 2. Examined gene-environment interaction. **Limitations**: No direct measure of folate exposure in utero.
**Roza, 2010** [16]	Cohort study, 4,214 toddlers followed at age of 18 months.	Folic acid supplementation was obtained by questionnaire at 18 weeks of gestation.	Beneficial. Children born to mothers with no folic acid supplement in the first trimester had a higher risk of total problems (OR 1·44; 95% CI 1·12, 1·86), internalising (OR 1·65; 95% CI 1·24, 2·19), and externalising problems (OR 1·45; 95% CI 1·17, 1·80), after adjusting for a number of confounding factors.	**Strengths**: 1. Large sample; 2. Prospective cohort design. **Limitations**: 1.Self-report of folic acid supplement during pregnancy; 2. Self-report of maternal behavioural problems psychopathology.
**Schlotz, 2010** [48]	Cohort study, 100 children followed to age of 8.75 years.	Maternal red blood cell (RBC) folate was measured at 14 weeks of gestation and folate intake from diet and supplement was assessed in early and late pregnancy.	Beneficial. Lower maternal RBC folate and folate intake in early pregnancy was associated with higher childhood hyperactivity (RCF: *beta* = −.24; *p* = .013; TFI: *beta* = −.24; *p* = .022) and peer problems scores (RCF: *beta* = −.28; *p* = .004; TFI: *beta* = −.28; *p* = .009). Adjustment for mother’s smoking and drinking alcohol during pregnancy did not change the results.	**Strengths**: Measurement of RBC folate and folate intake from food intake simultaneously. **Limitations**: 1. Small sample size; 2. Some important confounding factors were not available; 3. Outcomes assessed by mothers’ report.
**Veena, 2010** [49]	Cohort study, 536 children followed to age of 9–10 years.	Plasma folate was measured in samples taken at 30±2 weeks of gestation.	Beneficial. Children’s cognitive test scores increased by 0.1–0.2 SD per SD increase in maternal folate concentrations.	**Strengths**: 1. Cognitive function tests were specifically adapted for and validated in a South Indian population; 2. Adjusted for a number of important confounding factors. **Limitations**: 1. Maternal micronutrient assays were performed using plasma samples stored for 8y; 2. lack of data on maternal folate supplement at the time of blood sample collection; 3. Lack of information on parental IQ and the home environment.
**Campoy, 2011** [50]	Cohort study, 154 Children followed at age of 6.5 years.	Folate concentrations were determined in maternal blood 20 and 30 of gestation and at delivery, and in cord blood.	No association. No association between folate and cognitive function of children.	**Limitations**: Small sample size.
**Roth, 2011** [14]	Cohort study, 38,954 children followed to age of 3 years.	Folic acid supplement by questionnaire at 17 weeks of gestation.	Beneficial. Maternal folic acid supplementation showed a major protective effect on severe language delay. Children born to mothers who took no dietary supplement as the reference group (n = 9052 [24.0%], with severe language delay in 81 children [0.9%]). Adjusted ORs for 3 patterns of maternal dietary supplements were (1) other supplements, but no folic acid (n = 2480 [6.6%], with severe language delay in 22 children [0.9%]; OR, 1.04; 95% CI, 0.62–1.74); (2) folic acid only (n = 7127 [18.9%], with severe language delay in 28 children [0.4%]; OR, 0.55; 95% CI, 0.35–0.86); and (3) folic acid in combination with other supplements (n = 19,005 [50.5%], with severe language delay in 73 children [0.4%]; OR, 0.55; 95% CI, 0.39–0.78).	**Strengths**: 1. Large sample size; 2. Prospective cohort study design; 3. Used a solid outcome measure. **Limitations**: 1. Self-reported supplementation; 2. Residual confounding.
**Chatzi, 2012** [13]	Cohort study, 553 children followed to age of 18 months.	Folic acid supplementation by questionnaire at 14–18 weeks of gestation.	Beneficial. Compared with non-users, daily intake of 5 mg supplemental of folic acid was associated with a 5-unit increase on the scale of receptive communication and a 3.5-unit increase on the scale of expressive communication. Doses of folic acid supplementation higher than 5 mg/d were not associated with additional increase in the neurodevelopmental scales.	**Strengths**: 1. High participation rate (90%); 2. The Bayley-III that was used for the neurodevelopmental assessment of children. **Limitations**: 1. Self-reported supplementation; 2. Residual confounding.
**Forns, 2012** [12]	Cohort study, 393 children followed to age of 11 years.	Folic acid supplementation by structured interview at the end of the first trimester.	Beneficial. Omission errors were lower in those children whose mothers took dietary supplementation with folic acid during pregnancy.	**Limitations**: 1. Small sample size; 2. Self-report folic acid supplementation.
**Steenweg-de, 2012** [15]	Cohort study, 3,209 children followed to age of 3 years.	Maternal plasma folate concentrations measured and folic acid supplement collected (though questionnaire) in early pregnancy.	Beneficial. Children born to mothers with prenatal folate deficiency were at higher risk of emotional problems (OR: 1.57; 95% CI: 1.03, 2.38) but not behavioural problems (OR: 1.00; 95% CI: 0.64, 1.56) after adjustment for confounders. A higher risk of emotional problems was also found in children whose mothers started using folic acid supplements late or did not use supplements at all (OR: 1.45; 95% CI: 1.14, 1.84) than in children whose mothers started periconceptionally.	**Strengths**: 1. Large sample size; 2. Combination of data on plasma folate concentration, folic acid supplementation, and MTHFR genotype; 3. The ability to adjust for considerable numbers of covariates. **Limitations**: 1. Selective attrition may have influenced results; 2. Only one measure of maternal plasma folate concentration; 3. residual confounding.
**Villamor, 2012** [11]	Cohort study, 1,210 children followed to 3 years of age.	Folate intake by food frequency questionnaires.	Beneficial. In multivariable models adjusting for potential sociobehavioural and nutritional confounders, for each 600 mg/d increment in total folate intake during the first trimester, the Peabody Picture Vocabulary Test III score at age 3 years was 1.6 points [95% confidence interval (CI) 0.1, 3.1; *P* = 0.04] higher.	**Strengths**: 1. Large sample size; 2. Prospective cohort study design; 3. Controlled for major potential confounders. **Limitations**: 1. The reference amount of folate intake, 600 mg/day, was high; 2. Residual confounding.
**Wu, 2012** [51]	Cohort study, 154 children followed to age of 18 months.	Maternal plasma folate level was measured at 16 to 36 weeks of gestation;	No association. Maternal plasma folic acid was not associated with neurodevelopment.	**Limitations**: 1. Small sample size; 2. Short follow up time (to 18 months only); 3. Blood sample was taken in a wide range of gestation (from 16 to 36 weeks), creating large measurement variations and thus further diminishing the study power.
**Surén, 2013** [8]	Cohort study, 85,176 children followed at age of 3.3–10.2 years.	Folic acid supplementation in early pregnancy through questionnaire at 18 weeks of gestation.	Beneficial. In children born to mothers who took folic acid, 0.10% (64/61 042) had autistic disorder, compared with 0.21% (50/24 134) in children born to mothers who did not take folic acid. The adjusted OR for autistic disorder in children of folic acid users was 0.61 (95% CI, 0.41–0.90).	**Strengths**: 1. Large and national representative sample; 2. Prospective cohort study design; 3. The use of a combination of screening, referral, and registry linkage for detection of ASD; 4. The richness of the exposure data allowed for differentiations between different supplements and between the various stages of pregnancy. **Limitations:** 1. Self-report folic acid supplementation; 2. Residual confounding.
**Steenweg-de Graaff J, 2014** [52]	Cohort study, 3,893 children followed to age of 6 years.	Maternal plasma folate concentrations folic acid supplementation by questionnaire in early pregnancy.	Beneficial. Children born to mothers who started using folic acid supplements before conception had lower scores on autistic traits than children born to mother who did not use folic acid supplements.	**Strengths:** 1. Large sample size; 2. Prospective cohort study design; 3. Measured both maternal plasma folate and folic acid supplementation not. **Limitations:** Diagnosis of autism was not at the authors’ disposal.
**Valera-Gran D, 2014** [18]	Cohort study, 2,213 children followed to age of 7 years.	Pregnant women completed an interviewer-administered questionnaire on the usual dietary folate intake and FA supplements at 10 to 13 weeks and 28 to 32 weeks of gestation.	A high proportion of women (57.3%) did not reach the recommended dosages of FA supplements (400 μg/d), but 25.2% women took more than 1000 μg/d of FA supplements (3.5% consuming >5000 μg/d). In multivariate analysis, we observed that children whose mothers used FA supplement dosages higher than 5000 μg/d during pregnancy had a statistically significantly lower mean psychomotor scale score (difference, −4.35 points; 95% CI, −8.34 to −0.36) than children whose mothers used a recommended dosage of FA supplements (400–1000 μg/d). An increased risk of delayed psychomotor development (psychomotor scale score <85) was also evident among children whose mothers took FA supplement dosages higher than 5000 μg/d, although the association was not statistically significant (odds ratio = 1.59; 95% CI, 0.82–3.08).	**Strengths:** The multicenter structure of this study and the wide range of usual dietary folate intake and FA supplement patterns provide results that can be extrapolated to a wide range of situations. Moreover, the prospective cohort design of the INMA Project study allows for the control of a wide variety of confounding variables and for the blinded and homogeneous conditions in which the children of women using and not using FA supplements were evaluated. **Limitations:** We adjusted for a wide range of potential risk factors, although the effects of other potential confounding factors or modifiers cannot be discarded. Regarding FA supplement use, it can be argued that mothers with higher-risk pregnancy are more likely to be encouraged to take supplements. Another limitation may be that we did not account for maternal IQ and mental health, although we did adjust for sociodemographic covariates that may partly correct for them.
**Schmidt, 2011** [9]	Case control study, 707 children (24–60 months) diagnosed with autism, autism spectrum disorder, or normal development	Telephone interviews of prenatal vitamins (including folic acid) consumption at any time during the period three months before conception through pregnancy and the breastfeeding period.	Beneficial. Significant interaction effects were observed for maternal MTHFR 677 TT, CBS rs234715 GT+TT, and child COMT 472 AA genotypes, with greater risk for autism when mothers did not report taking prenatal vitamins periconceptionally (4.5 [1.4–14.6]; 2.6 [1.2–5.4]; and 7.2 [2.3–22.4], respectively). Greater risk was also observed for children whose mothers had other one-carbon metabolism pathway gene variants and reported no maternal prenatal vitamin intake.	**Strengths:** 1. Clinically confirmed diagnosis; 2. Controlled for a number of potential confounders; 3. Large sample size; 4. Assessed gene—environment interaction. **Limitations:** Retrospective reporting of supplementation of folic acid and other vitamins.
**Schmidt, 2012** [10]	Case control study, 837 children (20–60 months) diagnosed with autism, autism spectrum disorder, or normal development	Average daily folic acid was quantified for each mother on the basis of dose, brands, and intake frequency of vitamins, supplements, and breakfast cereals reported through structured telephone interviews.	Beneficial. Mean (±SEM) folic acid intake was significantly greater for mothers of normal children than for mothers of children with ASD in the first month of pregnancy (P1; 779.0±36.1 and 655.0±28.7 ug, respectively; P<0.01). A mean daily folic acid intake of ≥600 ug (compared with <600 ug) during pregnancy was associated with reduced ASD risk (adjusted OR: 0.62; 95% CI: 0.42, 0.92; P = 0.02), and risk estimates decreased with increased folic acid (P-trend = 0.001). The association between folic acid and reduced ASD risk was strongest for mothers and children with MTHFR 677 C>T variant genotypes.	**Strengths:** 1. Clinically confirmed diagnosis; 2. Controlled for a number of potential confounders; 3. Large sample size; 4. Assessed gene—environment interaction. **Limitations:** Retrospective reporting of supplementation of folic acid and other vitamins.

*Results were considered beneficial if they consistently showed better outcomes in the group with folic acid supplementation in pregnancy (or higher maternal folate level) than the comparison group. If study results consistently showed poorer outcomes in the group with folic acid supplementation in pregnancy (or higher maternal folate level) than in the comparison group these results were defined as harmful. Where there was no difference between (or among) comparison groups, or conflicting findings on different outcome measures (i.e. protective on one outcome but harmful on another, study results were defined as no association.

### Study characteristics

The 22 eligible studies include 2 RCTs, 18 cohort studies, and 2 case control studies. The baseline characteristics and further summarized information are outlined in Table 2. The main outcomes include ASDs, autism, developmental delay, cognition, attention function, neurodevelopment, emotional problems, and behavioural problems. The children range in age from 12 months to 11 years. Among the 21studies, 7 studies included more than 1000 children.

### Outcomes

Fifteen studies showed a beneficial effect of folic acid supplementation on neurodevelopment/autism, 6 studies found no statistically significant result, while one study found a harmful effect at high dose of folic acid supplementation (see Table 2). There were 3 studies that had ASD as the main outcome measure. The first was a cohort study of 85,176 children aged 3.3 to 10.2 years [8]. The rate of ASD in children whose mothers took folic acid was 0.10%, whereas the rate for mothers who did not take folic acid was 0.21%, with adjusted odds ratio (OR) of folic acid users 0.61 (95% confidence interval (CI), 0.41–0.90). Another study was the Childhood Autism Risks from Genetics and Environment, a case-control study in the United States [9]. In the 837 mother-child pairs, the mean folic acid intake in the first month of pregnancy was significantly greater for mothers of normally developing children than for mothers of children with a confirmed diagnosis of ASD. A mean daily folic acid intake of ≥ 600ug during the first pregnant month was associated with reduced ASD risk (adjusted OR: 0.62; 95% CI: 0.42–0.92; P = 0.02). This finding was consistent with another case-control study by the same author [10], which showed that mean folic acid intake in early pregnancy was significantly higher for mothers of normally developing children than for mothers of children with ASD.

Several studies found similar beneficial effects of folic acid supplementation on other areas of neurodevelopment. For example, a study in Massachusetts [11] showed that for each 600 ug/day increment in total folate intake during the first trimester, Peabody Picture Vocabulary Test-III score at age 3 years was 1.6 points (95% CI 0.1–3.1; p = 0.04) higher. Forns et al found that omission errors (defined as the number of targets to which the individual did not respond) were lower in those whose mothers took dietary supplementation with folic acid and vitamins during pregnancy [12]. In a cohort study [13] involving 553 mother-child pairs in Greece, neurodevelopment at 18 months was assessed using the Bayley Scales of Infant and Toddler Development (3rd edition). Compared with non-users, daily intake of 5 mg supplemental folic acid was associated with a 5-unit increase on the scale of receptive communication and a 3.5-unit increase on the scale of expressive communication. Roth et al assessed severe language delay (defined as minimal expressive language: only 1-word or unintelligible utterances at the age of 3 years) in a cohort of 38,954 children, and found that adjusted ORs for 3 patterns of exposure to maternal dietary supplements (no supplement as the reference) were 1.04 (95% CI, 0.62–1.74) for other supplements but no folic acid; 0.55 (95% CI, 0.35–0.86) for folic acid only; and 0.55 (95% CI, 0.39–0.78) for folic acid in combination with other supplements, demonstrating a clear protective effect of folic acid supplementation during pregnancy [14]. A study by Steenweg-de et al found a higher risk of emotional problems in 3 year old children using the Child Behavior Checklist (CBCL) whose mothers did not use supplements or started folic acid supplements late in pregnancy (OR: 1.45; 95% CI: 1.14, 1.84) compared to children whose mothers started folic acid supplement in early pregnancy [15]. Similarly, in a prospective cohort study, Roza et al examined the association between folic acid supplement use during the first trimester and behavioural and emotional problems identified by the CBCL in 4,214 toddlers at the age of 18 months. This study found that folic acid supplement use protected both from internalizing (OR of no use 1.65; 95% CI 1.24, 2.19) and externalizing problems (OR of no use 1.45; 95% CI 1.17, 1.80), after adjusting for maternal characteristics, birth weight, and fetal head size [16]. In another prospective cohort study, Julvez et al found that folic acid supplement during pregnancy was associated with improved neurodevelopment in children after adjusting for a number of sociodemographic and behavioural factors (results obtained from linear regression models): higher scores on verbal (b (regression slope) = 3.98, SE (standard error of regression slope) = 1.69), motor (b = 4.54, SE = 1.66), verbal-executive function (b = 3.97, SE = 1.68) scores, social competence (b = 3.97, SE = 1.61), and lower rate of inattention symptom [OR = 0.46; 95% CI 0.22, 0.95] [17].

## Discussion

A total of 22 original papers that looked at the association between folic acid supplementation in pregnancy and neurodevelopment/autism were identified after the screening, with 15 studies showing a beneficial effect of folic acid supplementation on neurodevelopment/autism, 6 studies found no statistically significant effect, while one study found a harmful effect at high dose of folic acid supplementation [18]. Two papers that suggested an adverse effect of folic acid on ASDs were not included in our review because no data on individual subjects were available in these two studies [19, 20]. Both papers used ecological data to support their hypothesis: prenatal folic acid supplementation and autism diagnoses in the United States since the 1980s in King’s study, and published autism incidence rates and prescriptions for folic acid in Rochester, Minnesota from 1976 to 1997 in Beard’s study. Beard’s study found a correlation coefficient of 0.87 (95% CI 0.19–0.99) between autism rates and the prescription prenatal vitamins containing folic acid and a correlation coefficient of 0.62 (95% CI 0.38–0.95) between autism rates and pediatric folic acid. However, during the same period, major changes in other factors such as diagnostic criteria, public awareness, disease surveillance, and screening efforts have all played an important role in the increased rates of diagnosed ASDs, so ecological data may not be suitable to analyze the association between folate and ASDs. Data from recent ASDs surveillance in the United States revealed a major increase in ASDs prevalence during a period with no change in policies regarding prenatal folic acid supplementation or folic acid food fortification (2002 to 2008), suggesting that ecological analyses are seriously flawed [21]. On the other hand, in a small sample of children (77) born to mothers used folic acid supplementation >5 mg/day during pregnancy had a statistically significantly lower mean psychomotor scale score (difference, -4.35 points; 95% CI, -8.34 to -0.36) than children whose mothers used a recommended dosage of folic acid supplements (0.4–1.0 mg/day) [18]. The finding from a single study with small sample needs to be replicated. Castro et al conducted a systematic review of studies involving on relationship between folic acid and ASD. All 11 papers included in Castro’s review met the inclusion criteria in our review. It concluded that although lower folate levels were associated with increased risk of ASD, the effects of folate-enhancing interventions on the clinical symptoms of ASD have yet to be confirmed [22].

Folic acid, or folate (vitamin B9) is an essential nutrient that is required for DNA replication and as a substrate for a range of enzymatic reactions involved in amino acid synthesis and vitamin metabolism. Demands for folate increase during pregnancy because of increased demands from the fetus. It has been conclusively established that folic acid deficiency prior to and during early pregnancy (up to 12 weeks of gestation) causes increased risk of NTDs, and periconceptional supplementation of folic acid can dramatically lower this risk (as much as 70%) [3, 4, 5, 6]. If folic acid deficiency prior to and during early pregnancy can cause NTDs, it may also cause milder forms of fetal brain damage that could be expressed as impaired neurodevelopment/autism in early childhood, and this effect may not be restricted to pre-conception and early gestation (<12 weeks of gestation, as the neural tube closes at that time so no NTDs after that). Laboratory investigations in animals and humans have shown that folate plays an important role in early brain development. In humans, there are active placental transports of folate and fetal brain folate levels are higher than adult levels [6]. In rats, the concentrations of many folate-dependent enzymes were substantially higher during early development than adult levels [23]. Dams and developing pups fed with diets eliminating folic acid 1 week prior to birth were less viable and had lower brain weights, lower activity level, and lower level of S-adenosyl-L-methionine concentrations in brain tissue of surviving offspring than animals reared on normal diets [24]. Ferguson et al examined whether gestational dietary folate deficiency not producing severe NTDs could lead to other functional impairments in mice [25]. They found that prenatal folate deficiency in mice led to an increase in anxiety-related behaviours. Worthy of our attention is that Padmanabhan et al found a hypomorphic mutation of the mouse MTRR gene, which results in developmental delay, as well as congenital malformations, including neural tube, heart, and placental defects, showing that folate metabolism has distinct transgenerational epigenetic functions that are responsible for specific developmental processes [26]. Even with normal dietary folate, the hypomorphic mutations in the MTRR gene associated with reduced expression may still lead to congenital abnormalities.

A few clinical studies have compared metabolites or cofactors of the folate-methionine pathway in children with autism [27–37]. While results from these studies have not been consistent a dysfunctional folate-methionine pathway has been identified that may have an impact on developmental delays including autism [38]. This pathway is crucial for DNA synthesis, DNA methylation, and cellular redox balance.

Clinical case series have also linked folic acid deficiency to other types of fetal brain damage such as intracranial calcification [39]. Del Campo et al reported several cases of patients who, in addition to the structural anomalies typical of maternal methotrexate exposure, have significant developmental delay, and suggested that prenatal exposure to folic acid antagonists increases the risk of mental retardation [40]. Arakawa et al observed that the EEG maturation was delayed in children born to mothers with low serum folate [41]. A recent study evaluated the nutritional intake in 111 Chinese children with autism (aged 2 to 9 years) and compared with the national Dietary Reference Intakes (DRI) [42]. They found that the children with autism had adequate or exceeded intakes in energy, protein, vitamins B1, B2, E, niacin, magnesium, and iron, but had inadequate intakes in folic acid, vitamins A, B6, C, calcium, and zinc, nutrients known to be important for brain development and function [42].

### Strengths

To our knowledge, this is the first systematic review examining the impact of folic acid supplementation during pregnancy on neurodevelopment/autism in the offspring children. We did an extensive search of relevant literature and selected studies strictly. After merging and analyzing the selected studies, we provided a preliminary conclusion.

### Limitations

Our study has some limitations. First, the magnitude of the protective effect of folic acid supplementation observed in most of the included studies was quite small as compared with the known effect of folic acid supplementation on NTDs. We speculate that compared with NTDs, the diagnosis of neurodevelopmental disorders is more subtle and requires a much longer observation period. As a result, the potential effect of folic acid supplementation may have been offset by measurement errors or loss of follow ups. Second, most of the included studies were observational. However, one RCT [43] showed beneficial effects of folic acid supplementation, consistent with a majority of the observational studies, which adds weight to the evidence. Third, because of major heterogeneity in original studies in terms of study design and outcome and exposure measurements, no attempt to summarize the effect by meta-analysis was made. Finally, there may be studies with negative results that were not published in the searchable databases because of potential publication bias. Although the major heterogeneity of the included studies prevented us from a formal assessment of publication bias, global inspection of all included studies did not find any systematic trends in terms of positive/negative findings.

### Implications for research

The limited data identified suggests that folic acid supplementation in pregnancy protects against impaired neurodevelopment including ASDs in children, and may improve cognitive function and intellectual and motor function. However, it is hard to draw a conclusion due to the limitations of the identified studies. Large scale RCTs with validated diagnosis and high follow up rate are needed in order to produce robust evidence regarding the effects of folic acid supplementation in pregnancy on fetal neurodevelopment.

## Conclusion

In summary, our review of the literature suggests that folic acid supplementation in pregnancy may protect against impaired neurodevelopment including ASDs in children, and may improve cognitive function, intellectual, and motor function.

## Supporting Information

S1 PRISMA Checklist PRISMA 2009 checklist. (DOC)

S1 Appendix List of full-text excluded articles with reasons of exclusion. (DOCX)
