# Supplementary material for: New Perspective on Impact of Folic Acid Supplementation during Pregnancy on Neurodevelopment/Autism in the Offspring Children – A Systematic Review
Source: PLoS One. 2016 Nov 22;11(11):e0165626. doi: 10.1371/journal.pone.0165626 (PMC5119728; doi:10.1371/journal.pone.0165626)
Supplement: S1 PRISMA Checklist — (DOC) [file pone.0165626.s001.doc]

| **Section/topic** | **#** | **Checklist item** | **Reported on page #** |
| --- | --- | --- | --- |
| **TITLE** | | |  |
| Title | 1 | New perspective on impact of folic acid supplementation during pregnancy on neurodevelopment/autism in the offspring children – a systematic review | 1 |
| **ABSTRACT** | | |  |
| Structured summary | 2 | It has been conclusively established that folic acid supplementation prior to and during early pregnancy (up to 12 weeks of gestation) can prevent neural tube defects (NTDs). We hypothesized that folate effects may extend from neuro-structural defects to alterations in neuro-behavioural and emotional skills including autism spectrum disorders (ASDs) and other developmental disorders. The objective of this review was to comprehensively evaluate evidence on the impact of folic acid on neurodevelopment other than NTDs. We conducted an online search of relevant literature compiled by the National Library of Medicine from Medline and EMBASE (searched on Dec 31, 2014: <http://www.ncbi.nlm.nih.gov/entrez/query/fcgi> and <http://www.elsevier.com/online-tools/embase>). We first created 3 files (search restricted to English literature) using the following key words: 1) folate or folic acid (171322 papers identified by this search); 2) maternal or pregnancy or pregnant or gestation or gestational or prenatal or antenatal or periconception or periconceptional (1349219 papers identified by this search); and 3) autism or autism spectrum disorders or developmental delay or development or neurodevelopment or mental or cognitive or language or personal-social or gross motor or fine motor or behaviour or intellectual or intelligence or Bayley Scale (8268145 papers identified by this search). We then merged the 3 files and reviewed the papers that addressed these three issues simultaneously. A total of 22 original papers that examined the association between folic acid supplementation in human pregnancy and neurodevelopment/autism were identified after the screening, with 15 studies showing a beneficial effect of folic acid supplementation on neurodevelopment/autism, 6 studies showed no statistically significant difference, while one study showed a harmful effect in > 5 g folic acid supplementation/day during pregnancy. Folic acid supplementation in pregnancy may have beneficial effects on the neurodevelopment of children beyond its proven effect on NTDs. | 2 |
| INTRODUCTION | | |  |
| Rationale | 3 | There are major emotional, societal, and economic implications of impaired neurodevelopment and/or  autism in children [1, 2]. These children will often require specialized schooling and other community resources. Although the survival/life-span of these infants may not be seriously affected, many of them may need treatments throughout their lifetime, and the cost to the public health care system could be huge. When they reach adulthood, productivity is often lower than those with normal development, indirectly increasing the societal burden. It has been established that supplementation with folic acid around the time of conception reduces the risk of neural tube defects (NTDs) in the offspring [3, 4, 5, 6]. However, whether folic acid has a similar effect on impaired neurodevelopment and/or autism remains elusive. | 3 |
| Objectives | 4 | This article therefore focuses on assessing the role of folic acid supplementation during pregnancy and folate metabolism on neurodevelopmental outcomes including autism spectrum disorders (ASDs), other than NTDs. | 3 |
| METHODS | | |  |
| Protocol and registration | 5 | We conducted an online search of relevant literature compiled by the National Library of Medicine from Medline and EMBASE (searched on December 31, 2014 of the site: <http://www.ncbi.nlm.nih.gov/entrez/query/fcgi> and <http://www.elsevier.com/online-tools/embase>), with restriction to human studies. | 3 |
| Eligibility criteria | 6 | We included randomized controlled trials (RCTs), cohort studies, and case control studies that examined the association between folic acid supplementation during pregnancy and neurodevelopment/autism in the offspring children. | 4 |
| Information sources | 7 | We conducted an online search of relevant literature compiled by the National Library of Medicine from Medline and EMBASE (searched on December 31, 2014 of the site: <http://www.ncbi.nlm.nih.gov/entrez/query/fcgi> and <http://www.elsevier.com/online-tools/embase>), with restriction to human studies. | 3 |
| Search | 8 | We first created 3 files (restricting our search to English literature) using the following key words: 1) folate or folic acid (171322 papers identified by this search); 2) maternal or pregnancy or pregnant or gestation or gestational or prenatal or antenatal or periconception or periconceptional (1349219 papers identified by this search); and 3) autism or autism spectrum disorders or developmental delay or development or neurodevelopment or mental or cognitive or language or personal-social or gross motor or fine motor or behaviour or intellectual or intelligence or Bayley Scale (8268145 papers identified by this search). We then merged the 3 files. All abstracts of the papers identified by merging the 3 files were screened by two independent reviewers in our group to exclude irrelevant studies (such as those on NTDs); because the causation between folic acid supplementation in pregnancy and NTDs has been established, our review is interested in outcomes other than NTDs. | 3-4 |
| Study selection | 9 | We included randomized controlled trials (RCTs), cohort studies, and case control studies that examined the association between folic acid supplementation during pregnancy and neurodevelopment/autism in the offspring children. | 4 |
| Data collection process | 10 | Two independent reviewers separately screened all records at the title level. To enhance sensitivity, records were only removed if both reviewers excluded at the title level. The second level of review was at the abstract level followed by another round of review at the full-text level. Two independent reviewers abstracted data using a standardized form. When there was a disagreement it was resolved by discussion with a third reviewer. Corresponding authors were contacted via e-mail at least three times to obtain data if the outcome of the neurodevelopment/autism in the offspring children could not be readily abstracted from the publication. | 4 |
| Data items | 11 | Data extracted from each study included the first author’s last name, publication year, main outcome, sample size, study design, age of children, effect of folic acid, and comments on the study. Because of major heterogeneity in original studies in terms of study design and outcome and exposure measurements, no attempt to summarize the effect by meta-analysis was made. When studies demonstrated conflicting findings on different outcome measures, the study was defined as “no association”. | 6 |
| Risk of bias in individual studies | 12 | The quality of included cohort and case control studies was assessed with the Newcastle Ottawa Scale [7].  Using this checklist, two reviewers evaluated each of the included articles. | 4 |
| Summary measures | 13 | risk ratio, confidence interval. | None |
| Synthesis of results | 14 | Because of major heterogeneity in original studies in terms of study design and outcome and exposure measurements, no attempt to summarize the effect by meta-analysis was made. When studies demonstrated conflicting findings on different outcome measures, the study was defined as “no association”. | 6 |

Page 1 of 2

| **Section/topic** | **#** | **Checklist item** | **Reported on page #** |
| --- | --- | --- | --- |
| Risk of bias across studies | 15 | publication bias | None |
| Additional analyses | 16 | none | None |
| **RESULTS** | | |  |
| Study selection | 17 | A total of 1915 papers were identified by merging all 3 files. Many of these papers were either animal studies or review papers. Most frequently, in original studies in humans that did include all three of our focus areas, (folic acid supplementation, pregnancy, and neurodevelopment/autism in offspring), the effects of folic acid supplementation during pregnancy on neurodevelopment/autism in the offspring children was secondary, discussing this relationship in the interpretation of their study findings on other pregnancy outcomes (e.g., NTDs). | 6 |
| Study characteristics | 18 | The 22 eligible studies include 2 RCTs, 18 cohort studies, and 2 case control studies. The baseline characteristics and further summarized information are outlined in Table 2. The main outcomes include ASDs, autism, developmental delay, cognition, attention function, neurodevelopment, emotional problems, and behavioural problems. The children range in age from 12 months to 11 years. Among the 21studies, 7 studies included more than 1000 children. | 11 |
| Risk of bias within studies | 19 | A total of 22 original papers that looked at the association between folic acid supplementation in pregnancy and neurodevelopment/autism were identified after the screening (Table 2).After merging and analyzing the selected studies, we provided a preliminary conclusion. | 6 |
| Results of individual studies | 20 | A total of 22 original papers that looked at the association between folic acid supplementation in pregnancy and neurodevelopment/autism were identified after the screening (Table 2). | 6 |
| Synthesis of results | 21 | 15 studies showed a beneficial effect of folic acid supplementation on neurodevelopment/autism, 6 studies found no statistically significant result, while one study found a harmful effect at high dose of folic acid supplementation (see Table 2). There were 3 studies that had ASD as the main outcome measure. The first was a cohort study of 85,176 children aged 3.3 to 10.2 years [8]. The rate of ASD in children whose mothers took folic acid was 0.10%, whereas the rate for mothers who did not take folic acid was0.21%, with adjusted odds ratio (OR) of folic acid users 0.61 (95% confidence interval (CI), 0.41-0.90). Another study was the Childhood Autism Risks from Genetics and Environment, a case-control study in the United States [9]. In the 837 mother-child pairs, the mean folic acid intake in the first month of pregnancy was significantly greater for mothers of normally developing children than for mothers of children with a confirmed diagnosis of ASD. A mean daily folic acid intake of ≥ 600ug during the first pregnant month was associated with reduced ASD risk (adjusted OR: 0.62; 95% CI: 0.42-0.92; P=0.02). This finding was consistent with another case-control study by the same author [10], which showed that mean folic acid intake in early pregnancy was significantly higher for mothers of normally developing children than for mothers of children with ASD.  Several studies found similar beneficial effects of folic acid supplementation on other areas of neurodevelopment. For example, a study in Massachusetts [11] showed that for each 600 ug/day increment in total folate intake during the first trimester, Peabody Picture Vocabulary Test-III score at age 3 years was 1.6 points (95% CI 0.1-3.1; p=0.04) higher. Forns et al found that omission errors (defined as the number of targets to which the individual did not respond) were lower in those whose mothers took dietary supplementation with folic acid and vitamins during pregnancy [12]. In a cohort study [13] involving 553 mother-child pairs in Greece, neurodevelopment at 18 months was assessed using the Bayley Scales of Infant and Toddler Development (3rd edition). Compared with non-users, daily intake of 5 mg supplemental folic acid was associated with a 5-unit increase on the scale of receptive communication and a 3.5-unit increase on the scale of expressive communication. Roth et al assessed severe language delay (defined as minimal expressive language: only 1-word or unintelligible utterances at the age of 3 years) in a cohort of 38,954 children, and found that adjusted ORs for 3 patterns of exposure to maternal dietary supplements (no supplement as the reference) were 1.04 (95% CI, 0.62-1.74) for other supplements but no folic acid; 0.55 (95% CI, 0.35-0.86) for folic acid only; and 0.55 (95% CI, 0.39-0.78) for folic acid in combination with other supplements, demonstrating a clear protective effect of folic acid supplementation during pregnancy [14]. A study by Steenweg-de et al found a higher risk of emotional problems in 3 year old children using the Child Behavior Checklist (CBCL) whose mothers did not use supplements or started folic acid supplements late in pregnancy (OR: 1.45; 95% CI: 1.14, 1.84) compared to children whose mothers started folic acid supplement in early pregnancy [15]. Similarly, in a prospective cohort study, Roza et al examined the association between folic acid supplement use during the first trimester and behavioural and emotional problems identified by the CBCL in 4,214 toddlers at the age of 18 months. This study found that folic acid supplement use protected both from internalizing (OR of no use 1.65; 95 % CI 1.24, 2.19) and externalizing problems (OR of no use 1.45; 95 % CI 1.17, 1.80), after adjusting for maternal characteristics, birth weight, and fetal head size [16]. In another prospective cohort study, Julvez et al found that folic acid supplement during pregnancy was associated with improved neurodevelopment in children after adjusting for a number of sociodemographic and behavioural factors: higher scores on verbal (b = 3.98, SE = 1.69), motor (b = 4.54, SE = 1.66), verbal-executive function (b = 3.97, SE = 1.68) scores, social competence (b = 3.97, SE = 1.61), and lower rate of inattention symptom [OR = 0.46; 95% CI 0.22, 0.95] [17]. | 11-13 |
| Risk of bias across studies | 22 | After merging and analyzing the selected studies, we provided a preliminary conclusion. | None |
| Additional analysis | 23 | None | None |
| **DISCUSSION** | | |  |
| Summary of evidence | 24 | A total of 22 original papers that examined the association between folic acid supplementation in human pregnancy and neurodevelopment/autism were identified after the screening, with 15 studies showing a beneficial effect of folic acid supplementation on neurodevelopment/autism, 6 studies showed no statistically significant difference, while one study showed a harmful effect in > 5 g folic acid supplementation/day during pregnancy. Folic acid supplementation in pregnancy may have beneficial effects on the neurodevelopment of children beyond its proven effect on NTDs.  To our knowledge, this is the first systematic review examining the impact of folic acid supplementation during pregnancy on neurodevelopment/autism in the offspring children. We did an extensive search of relevant literature and selected studies strictly. After merging and analyzing the selected studies, we provided a preliminary conclusion. | 13 |
| Limitations | 25 | Our study has some limitations. First, the magnitude of the protective effect of folic acid supplementation observed in most of the included studies was quite small as compared with the known effect of folic acid supplementation on NTDs. We speculate that compared with NTDs, the diagnosis of neurodevelopmental disorders is more subtle and requires a much longer observation period. As a result, the potential effect of folic acid supplementation may have been offset by measurement errors or loss of follow ups. Second, most of the included studies were observational. However, one RCT [48] showed beneficial effects of folic acid supplementation, consistent with a majority of the observational studies, which adds weight to the evidence. Third, because of major heterogeneity in original studies in terms of study design and outcome and exposure measurements, no attempt to summarize the effect by meta-analysis was made. Finally, there may be studies with negative results that were not published in the searchable databases because of potential publication bias. Although the major heterogeneity of the included studies prevented us from a formal assessment of publication bias, global inspection of all included studies did not find any systematic trends in terms of positive/negative findings. | 15-16 |
| Conclusions | 26 | In summary, our review of the literature suggests that folic acid supplementation in pregnancy may protect against impaired neurodevelopment including ASDs in children, and may improve cognitive function, intellectual, and motor function. | 16 |
| **FUNDING** | | |  |
| Funding | 27 | This study was funded by the Canadian Institutes for Health Research (CIHR) (MOP-142723). | 19 |

*From:*  Moher D, Liberati A, Tetzlaff J, Altman DG, The PRISMA Group (2009). Preferred Reporting Items for Systematic Reviews and Meta-Analyses: The PRISMA Statement. PLoS Med 6(7): e1000097. doi:10.1371/journal.pmed1000097

For more information, visit: **www.prisma-statement.org**.

Page 2 of 2
