# Supplementary material for: New Perspective on Impact of Folic Acid Supplementation during Pregnancy on Neurodevelopment/Autism in the Offspring Children – A Systematic Review
Source: PLoS One. 2016 Nov 22;11(11):e0165626. doi: 10.1371/journal.pone.0165626 (PMC5119728; doi:10.1371/journal.pone.0165626)
Supplement: S1 Appendix — (DOCX) [file pone.0165626.s002.docx]

1.[Eur J Paediatr Neurol.](http://www.ncbi.nlm.nih.gov/pubmed/?term=Pregnancy+and+neurodevelopmental+outcomes+with+in-utero+antiepileptic+agent+exposure.+A+pilot+study.) 2015 Jan;19(1):37-40. doi: 10.1016/j.ejpn.2014.09.006. Epub 2014 Oct 8.

Pregnancy and neurodevelopmental outcomes with in-utero antiepileptic agent exposure. A pilot study.

[Arkilo D](http://www.ncbi.nlm.nih.gov/pubmed/?term=Arkilo%20D%5BAuthor%5D&cauthor=true&cauthor_uid=25457510)1, [Hanna J](http://www.ncbi.nlm.nih.gov/pubmed/?term=Hanna%20J%5BAuthor%5D&cauthor=true&cauthor_uid=25457510)2, [Dickens D](http://www.ncbi.nlm.nih.gov/pubmed/?term=Dickens%20D%5BAuthor%5D&cauthor=true&cauthor_uid=25457510)2, [Justesen L](http://www.ncbi.nlm.nih.gov/pubmed/?term=Justesen%20L%5BAuthor%5D&cauthor=true&cauthor_uid=25457510)3, [Brunn J](http://www.ncbi.nlm.nih.gov/pubmed/?term=Brunn%20J%5BAuthor%5D&cauthor=true&cauthor_uid=25457510)3, [Garland S](http://www.ncbi.nlm.nih.gov/pubmed/?term=Garland%20S%5BAuthor%5D&cauthor=true&cauthor_uid=25457510)3, [Penovich P](http://www.ncbi.nlm.nih.gov/pubmed/?term=Penovich%20P%5BAuthor%5D&cauthor=true&cauthor_uid=25457510)2.

[Author information](http://www.ncbi.nlm.nih.gov/pubmed/?term=Pregnancy+and+neurodevelopmental+outcomes+with+in-utero+antiepileptic+agent+exposure.+A+pilot+study.)

Abstract

OBJECTIVE:

To assess pregnancy outcomes on women exposed to monotherapy with antiepileptic agents.

METHODS:

Questionnaires were sent to women with epilepsy in our practice who were pregnant between 2006 and 2011. 62/86 patients (72%) who responded were on monotherapy. 24 fetuses (63%) were exposed to lamotrigine, 11 (28%) to levetiracetam, 2 (5.2%) to topiramate, 1 (2.6%) to gabapentin, 17 (27%) to carbamazepine, 5 to phenytoin and 2 to valproate.

RESULTS:

There were 55 (88%) live births and 7 unsuccessful pregnancies (miscarriages/stillbirths). Unsuccessful pregnancies were reported in 2/24 gestations exposed to lamotrigine, 2/11 to levetiracetam and 3/17 to carbamazepine. Delayed motor development or speech delay requiring therapy and special programming was noted in 2/24 children prenatally exposed to lamotrigine, 3/17 exposed to carbamazepine and 1/2 children exposed to valproate.

CONCLUSION:

Our pilot study of children exposed to antiepileptic drug monotherapy in-utero demonstrated a favorable trend for successful pregnancy outcomes and developmental trajectory.

Copyright © 2014 European Paediatric Neurology Society. Published by Elsevier Ltd. All rights reserved.

KEYWORDS:

Antiepileptic medication; Developmental outcomes; Pregnancy outcomes

Exclusion reasons: No folic acid data

2. [Am J Clin Nutr.](http://www.ncbi.nlm.nih.gov/pubmed/?term=Improving+women%27s+diet+quality+preconceptionally+and+during+gestation%3A+effects+on+birth+weight+and+prevalence+of+low+birth+weight--a+randomized+controlled+efficacy+trial+in+India) 2014 Nov;100(5):1257-68. doi: 10.3945/ajcn.114.084921. Epub 2014 Sep 17.

Improving women's diet quality preconceptionally and during gestation: effects on birth weight and prevalenceof low birth weight--a randomized controlled efficacy trial in India (Mumbai Maternal Nutrition Project).

[Potdar RD](http://www.ncbi.nlm.nih.gov/pubmed/?term=Potdar%20RD%5BAuthor%5D&cauthor=true&cauthor_uid=25332324)1, [Sahariah SA](http://www.ncbi.nlm.nih.gov/pubmed/?term=Sahariah%20SA%5BAuthor%5D&cauthor=true&cauthor_uid=25332324)1, [Gandhi M](http://www.ncbi.nlm.nih.gov/pubmed/?term=Gandhi%20M%5BAuthor%5D&cauthor=true&cauthor_uid=25332324)1, [Kehoe SH](http://www.ncbi.nlm.nih.gov/pubmed/?term=Kehoe%20SH%5BAuthor%5D&cauthor=true&cauthor_uid=25332324)1, [Brown N](http://www.ncbi.nlm.nih.gov/pubmed/?term=Brown%20N%5BAuthor%5D&cauthor=true&cauthor_uid=25332324)1, [Sane H](http://www.ncbi.nlm.nih.gov/pubmed/?term=Sane%20H%5BAuthor%5D&cauthor=true&cauthor_uid=25332324)1, [Dayama M](http://www.ncbi.nlm.nih.gov/pubmed/?term=Dayama%20M%5BAuthor%5D&cauthor=true&cauthor_uid=25332324)1, [Jha S](http://www.ncbi.nlm.nih.gov/pubmed/?term=Jha%20S%5BAuthor%5D&cauthor=true&cauthor_uid=25332324)1, [Lawande A](http://www.ncbi.nlm.nih.gov/pubmed/?term=Lawande%20A%5BAuthor%5D&cauthor=true&cauthor_uid=25332324)1, [Coakley PJ](http://www.ncbi.nlm.nih.gov/pubmed/?term=Coakley%20PJ%5BAuthor%5D&cauthor=true&cauthor_uid=25332324)1, [Marley-Zagar E](http://www.ncbi.nlm.nih.gov/pubmed/?term=Marley-Zagar%20E%5BAuthor%5D&cauthor=true&cauthor_uid=25332324)1, [Chopra H](http://www.ncbi.nlm.nih.gov/pubmed/?term=Chopra%20H%5BAuthor%5D&cauthor=true&cauthor_uid=25332324)1,[Shivshankaran D](http://www.ncbi.nlm.nih.gov/pubmed/?term=Shivshankaran%20D%5BAuthor%5D&cauthor=true&cauthor_uid=25332324)1, [Chheda-Gala P](http://www.ncbi.nlm.nih.gov/pubmed/?term=Chheda-Gala%20P%5BAuthor%5D&cauthor=true&cauthor_uid=25332324)1, [Muley-Lotankar P](http://www.ncbi.nlm.nih.gov/pubmed/?term=Muley-Lotankar%20P%5BAuthor%5D&cauthor=true&cauthor_uid=25332324)1, [Subbulakshmi G](http://www.ncbi.nlm.nih.gov/pubmed/?term=Subbulakshmi%20G%5BAuthor%5D&cauthor=true&cauthor_uid=25332324)1, [Wills AK](http://www.ncbi.nlm.nih.gov/pubmed/?term=Wills%20AK%5BAuthor%5D&cauthor=true&cauthor_uid=25332324)1, [Cox VA](http://www.ncbi.nlm.nih.gov/pubmed/?term=Cox%20VA%5BAuthor%5D&cauthor=true&cauthor_uid=25332324)1, [Taskar V](http://www.ncbi.nlm.nih.gov/pubmed/?term=Taskar%20V%5BAuthor%5D&cauthor=true&cauthor_uid=25332324)1, [Barker DJ](http://www.ncbi.nlm.nih.gov/pubmed/?term=Barker%20DJ%5BAuthor%5D&cauthor=true&cauthor_uid=25332324)1, [Jackson AA](http://www.ncbi.nlm.nih.gov/pubmed/?term=Jackson%20AA%5BAuthor%5D&cauthor=true&cauthor_uid=25332324)1, [Margetts BM](http://www.ncbi.nlm.nih.gov/pubmed/?term=Margetts%20BM%5BAuthor%5D&cauthor=true&cauthor_uid=25332324)1, [Fall CH](http://www.ncbi.nlm.nih.gov/pubmed/?term=Fall%20CH%5BAuthor%5D&cauthor=true&cauthor_uid=25332324)1.

[Author information](http://www.ncbi.nlm.nih.gov/pubmed/?term=Improving+women%27s+diet+quality+preconceptionally+and+during+gestation%3A+effects+on+birth+weight+and+prevalence+of+low+birth+weight--a+randomized+controlled+efficacy+trial+in+India)

Abstract

BACKGROUND:

Low birth weight (LBW) is an important public health problem in undernourished populations.

OBJECTIVE:

We tested whether improving women's dietary micronutrient quality before conception and throughout pregnancy increases birthweight in a high-risk Indian population.

DESIGN:

The study was a nonblinded, individually randomized controlled trial. The intervention was a daily snack made from green leafy vegetables, fruit, and milk (treatment group) or low-micronutrient vegetables (potato and onion) (control group) from ≥ 90 d before pregnancy until delivery in addition to the usual diet. Treatment snacks contained 0.69 MJ of energy (controls: 0.37 MJ) and 10-23% of WHO Reference Nutrient Intakes of β-carotene, riboflavin, folate, vitamin B-12, calcium, and iron (controls: 0-7%). The primary outcome was birth weight.

RESULTS:

Of 6513 women randomly assigned, 2291 women became pregnant, 1962 women delivered live singleton newborns, and 1360 newborns were measured. In an intention-to-treat analysis, there was no overall increase in birth weight in the treatment group (+26 g; 95% CI: -15, 68 g; P = 0.22). There was an interaction (P < 0.001) between the allocation group and maternal prepregnant body mass index (BMI; in kg/m(2)) [birth-weight effect: -23, +34, and +96 g in lowest (<18.6), middle (18.6-21.8), and highest (>21.8) thirds of BMI, respectively]. In 1094 newborns whose mothers started supplementation ≥ 90 d before pregnancy (per-protocol analysis), birth weight was higher in the treatment group (+48 g; 95% CI: 1, 96 g; P = 0.046). Again, the effect increased with maternal BMI (-8, +79, and +113 g; P-interaction = 0.001). There were similar results for LBW (intention-to-treat OR: 0.83; 95% CI: 0.66, 1.05; P = 0.10; per-protocol OR = 0.76; 95% CI: 0.59, 0.98; P = 0.03) but no effect on gestational age in either analysis.

CONCLUSIONS:

A daily snack providing additional green leafy vegetables, fruit, and milk before conception and throughout pregnancy had no overall effect on birth weight. Per-protocol and subgroup analyses indicated a possible increase in birth weight if the mother was supplemented ≥ 3 mo before conception and was not underweight. This trial was registered at www.controlled-trials.com/isrctn/ as ISRCTN62811278.

Exclusion reasons: No autism data

3. [Tijdschr Psychiatr.](http://www.ncbi.nlm.nih.gov/pubmed/25327347) 2014;56(10):660-7.

# [The influence of genes and environment on the development of autism spectrum disorders].

[Article in Dutch]

[Spek AA](http://www.ncbi.nlm.nih.gov/pubmed/?term=Spek%20AA%5BAuthor%5D&cauthor=true&cauthor_uid=25327347).

### Abstract

#### BACKGROUND:

Autism spectrum disorders (asd) occur in 0,6% of the general population. On the basis of research in the 70s, 80s and 90s many experts came to the conclusion that asd were in fact genetically determined and were linked only slightly to environmental factors. Recent research, however, indicates that environmental factors really do play an important role.

#### AIM:

To describe and identify the main trends in current research into the causes of asd.

#### METHOD:

The literature was studied with the help of Medline, Cochrane, Web of Science and ScienceDirect.

#### RESULTS:

Recent studies indicate that there is an underlying genetic cause in 35 to 60% of the cases of asd. Environmental factors play a greater role than previously thought and trigger the development of asd in people with a genetic vulnerability to asd. Not only is there evidence of risk factors for asd, but there is also evidence that certain factors protect against asd, for instance the use of folic acid before and during pregnancy.

#### CONCLUSION:

asd are probably related to a combination of gene mutations and environmental factors and to the interactions between the two. Further research is needed into the genetic and environmental causes of asd.

Exclusion reasons: Review

4. [Nutr Neurosci.](http://www.ncbi.nlm.nih.gov/pubmed/25087906) 2014 Aug 2. [Epub ahead of print]

Folic acid and autism: What do we know?

[Castro K](http://www.ncbi.nlm.nih.gov/pubmed/?term=Castro%20K%5BAuthor%5D&cauthor=true&cauthor_uid=25087906), [Klein LD](http://www.ncbi.nlm.nih.gov/pubmed/?term=Klein%20LD%5BAuthor%5D&cauthor=true&cauthor_uid=25087906), [Baronio D](http://www.ncbi.nlm.nih.gov/pubmed/?term=Baronio%20D%5BAuthor%5D&cauthor=true&cauthor_uid=25087906), [Gottfried C](http://www.ncbi.nlm.nih.gov/pubmed/?term=Gottfried%20C%5BAuthor%5D&cauthor=true&cauthor_uid=25087906), [Riesgo R](http://www.ncbi.nlm.nih.gov/pubmed/?term=Riesgo%20R%5BAuthor%5D&cauthor=true&cauthor_uid=25087906), [Perry IS](http://www.ncbi.nlm.nih.gov/pubmed/?term=Perry%20IS%5BAuthor%5D&cauthor=true&cauthor_uid=25087906).

Abstract

Autism spectrum disorders (ASD) consist in a range of neurodevelopmental conditions that share common features with autism, such as impairments in communication and social interaction, repetitive behaviors, stereotypies, and a limited repertoire of interests and activities. Some studies have reported that folic acid supplementation could be associated with a higher incidence of autism, and therefore, we aimed to conduct a systematic review of studies involving relationships between this molecule and ASD. The MEDLINE database was searched for studies written in English which evaluated the relationship between autism and folate. The initial search yielded 60 potentially relevant articles, of which 11 met the inclusion criteria. The agreement between reviewers was κ = 0.808. The articles included in the present study addressed topics related to the prescription of vitamins, the association between folic acid intake/supplementation during pregnancy and the incidence of autism, food intake, and/or nutrient supplementation in children/adolescents with autism, the evaluation of serum nutrient levels, and nutritional interventions targeting ASD. Regarding our main issue, namely the effect of folic acid supplementation, especially in pregnancy, the few and contradictory studies present inconsistent conclusions. Epidemiological associations are not reproduced in most of the other types of studies. Although some studies have reported lower folate levels in patients with ASD, the effects of folate-enhancing interventions on the clinical symptoms have yet to be confirmed.

KEYWORDS:

Autistic disorder; Folic acid; Vitamins

Exclusion reasons: Review

5. [ISRN Nutr.](http://www.ncbi.nlm.nih.gov/pubmed/?term=Increasing+prevalence%2C+changes+in+diagnostic+criteria%2C+and+nutritional+risk+factors+for+autism+spectrum+disorders.) 2014 Feb 13;2014:514026. doi: 10.1155/2014/514026. eCollection 2014.

Increasing prevalence, changes in diagnostic criteria, and nutritional risk factors for autism spectrum disorders.

[Neggers YH](http://www.ncbi.nlm.nih.gov/pubmed/?term=Neggers%20YH%5BAuthor%5D&cauthor=true&cauthor_uid=24967269)1.

[Author information](http://www.ncbi.nlm.nih.gov/pubmed/?term=Increasing+prevalence%2C+changes+in+diagnostic+criteria%2C+and+nutritional+risk+factors+for+autism+spectrum+disorders.)

Abstract

The frequency of autism spectrum disorders (ASD) diagnoses has been increasing for decades, but researchers cannot agree on whether the trend is a result of increased awareness, improved detection, expanding definition, or an actual increase in incidence or a combination of thesefactors. Though both genetic and multiple environmental risk factors have been studied extensively, many potentially modifiable risk factorsincluding nutritional and immune function related risk factors such as vitamin D, folic acid, and metabolic syndrome have not received sufficient attention. Several recent studies have put forward hypotheses to explain the mechanism of association between both folic acid and vitamin D andautism. A continuous rise in the prevalence of autism in the USA has coincided with a significant enhancement of maternal folate status with FDA mandated folic acid fortification of certain foods starting in 1998. There is also a growing body of research that suggests that vitamin D status either in utero or early in life may be a risk for autism. In this communication, controversies regarding increase in estimate of prevalence, implications ofchanges in definition, and possible association between some modifiable nutritional risk factors such as folic acid and vitamin D and ASD will be discussed.

Exclusion reasons: Review

6. Dev Med Child Neurol. 2014 Jul;56(7):604. doi: 10.1111/dmcn.12498.

Valproate and folic acid in pregnancy: associations with autism.

Baxter P.

Exclusion reasons: Review

7. [Int J Epidemiol.](http://www.ncbi.nlm.nih.gov/pubmed/24518932) 2014 Apr;43(2):443-64. doi: 10.1093/ije/dyt282. Epub 2014 Feb 11.

Maternal lifestyle and environmental risk factors for autism spectrum disorders.

[Lyall K](http://www.ncbi.nlm.nih.gov/pubmed/?term=Lyall%20K%5BAuthor%5D&cauthor=true&cauthor_uid=24518932)1, [Schmidt RJ](http://www.ncbi.nlm.nih.gov/pubmed/?term=Schmidt%20RJ%5BAuthor%5D&cauthor=true&cauthor_uid=24518932), [Hertz-Picciotto I](http://www.ncbi.nlm.nih.gov/pubmed/?term=Hertz-Picciotto%20I%5BAuthor%5D&cauthor=true&cauthor_uid=24518932).

[Author information](http://www.ncbi.nlm.nih.gov/pubmed/24518932)

Abstract

BACKGROUND:

Over the past 10 years, research into environmental risk factors for autism has grown dramatically, bringing evidence that an array of non-genetic factors acting during the prenatal period may influence neurodevelopment.

METHODS:

This paper reviews the evidence on modifiable preconception and/or prenatal factors that have been associated, in some studies, with autism spectrum disorder (ASD), including nutrition, substance use and exposure to environmental agents. This review is restricted to human studies with at least 50 cases of ASD, having a valid comparison group, conducted within the past decade and focusing on maternallifestyle or environmental chemicals.

RESULTS:

Higher maternal intake of certain nutrients and supplements has been associated with reduction in ASD risk, with the strongest evidence for periconceptional folic acid supplements. Although many investigations have suggested no impact of maternal smoking and alcohol use on ASD, more rigorous exposure assessment is needed. A number of studies have demonstrated significant increases in ASD risk with estimated exposure to air pollution during the prenatal period, particularly for heavy metals and particulate matter. Little research has assessed other persistent and non-persistent organic pollutants in association with ASD specifically.

CONCLUSIONS:

More work is needed to examine fats, vitamins and other maternal nutrients, as well as endocrine-disrupting chemicals and pesticides, in association with ASD, given sound biological plausibility and evidence regarding other neurodevelopmental deficits. The field can be advanced by large-scale epidemiological studies, attention to critical aetiological windows and how these vary by exposure, and use of biomarkers and other means to understand underlying mechanisms.

KEYWORDS:

Autism; air pollution; environmental chemicals; environmental risk factors; maternal alcohol use; maternal nutrition; maternal smoking

Exclusion reasons: Review

8. [JAMA Neurol.](http://www.ncbi.nlm.nih.gov/pubmed/?term=Early+child+development+and+exposure+to+antiepileptic+drugs+prenatally+and+through+breastfeeding%3A+a+prospective+cohort+study+on+children+of+women+with+epilepsy.) 2013 Nov;70(11):1367-74. doi: 10.1001/jamaneurol.2013.4290.

Early child development and exposure to antiepileptic drugs prenatally and through breastfeeding: aprospective cohort study on children of women with epilepsy.

[Veiby G](http://www.ncbi.nlm.nih.gov/pubmed/?term=Veiby%20G%5BAuthor%5D&cauthor=true&cauthor_uid=24061295)1, [Engelsen BA](http://www.ncbi.nlm.nih.gov/pubmed/?term=Engelsen%20BA%5BAuthor%5D&cauthor=true&cauthor_uid=24061295), [Gilhus NE](http://www.ncbi.nlm.nih.gov/pubmed/?term=Gilhus%20NE%5BAuthor%5D&cauthor=true&cauthor_uid=24061295).

[Author information](http://www.ncbi.nlm.nih.gov/pubmed/?term=Early+child+development+and+exposure+to+antiepileptic+drugs+prenatally+and+through+breastfeeding%3A+a+prospective+cohort+study+on+children+of+women+with+epilepsy.)

Abstract

IMPORTANCE:

Exposure to antiepileptic drugs during pregnancy is associated with adverse effects on psychomotor development.

OBJECTIVES:

To determine whether signs of impaired development appear already during the first months of life in children exposed prenatallyto antiepileptic drugs, and to explore potential adverse effects of antiepileptic drug exposure through breastfeeding.

DESIGN, SETTING, AND PARTICIPANTS:

Mothers at 13 to 17 weeks of pregnancy were recruited in the population-based, prospective Norwegian Mother and Child Cohort Study from 1999 to 2009. The mothers reported on their child's motor and social skills, language, and behavior using items from standardized screening tools at 6 months (n = 78,744), 18 months (n = 61,351), and 36 months (n = 44,147) of age. The mothers also provided detailed information on breastfeeding during the first year. MAIN OUTCOMES AND MEASURES The risk of adverse development in children according to maternal or paternal epilepsy was estimated as the odds ratio with corresponding 95% confidence interval, adjusted for maternal age, parity, education, smoking, breastfeeding, depression/anxiety, folate supplementation, and congenital malformation in the child.

RESULTS:

At age 6 months, infants of mothers using antiepileptic drugs (n = 223) had a higher risk of impaired fine motor skills compared with the reference group (11.5% vs 4.8%, respectively; odds ratio = 2.1; 95% CI, 1.3-3.2). Use of multiple antiepileptic drugs compared with the reference group was associated with adverse outcome for both fine motor skills (25.0% vs 4.8%, respectively; odds ratio = 4.3; 95% CI, 2.0-9.1) and social skills (22.5% vs 10.2%, respectively; odds ratio = 2.6; 95% CI, 1.2-5.5). Continuous breastfeeding in children of women usingantiepileptic drugs was associated with less impaired development at ages 6 and 18 months compared with those with no breastfeeding orbreastfeeding for less than 6 months. At 36 months, prenatal antiepileptic drug exposure was associated with adverse development regardless of breastfeeding status during the first year. Children of women with epilepsy who did not use antiepileptic drugs and children of fathers with epilepsy had normal development at 6 months.

CONCLUSIONS AND RELEVANCE:

Prenatal exposure to antiepileptic drugs was associated with impaired fine motor skills already at age 6 months, especially when the child was exposed to multiple drugs. There were no harmful effects of breastfeeding. Women with epilepsy should be encouraged to breastfeed their children irrespective of antiepileptic drug treatment.

Exclusion reasons: No folic acid data

9. [BJOG.](http://www.ncbi.nlm.nih.gov/pubmed/?term=Maternal+and+health+care+determinants+of+preconceptional+use+of+folic+acid+supplementation+in+France%3A+results+from+the+2010+National+Perinatal+Survey) 2013 Dec;120(13):1661-7. doi: 10.1111/1471-0528.12414. Epub 2013 Sep 10.

Maternal and health care determinants of preconceptional use of folic acid supplementation in France: resultsfrom the 2010 National Perinatal Survey.

[Tort J](http://www.ncbi.nlm.nih.gov/pubmed/?term=Tort%20J%5BAuthor%5D&cauthor=true&cauthor_uid=24034718)1, [Lelong N](http://www.ncbi.nlm.nih.gov/pubmed/?term=Lelong%20N%5BAuthor%5D&cauthor=true&cauthor_uid=24034718), [Prunet C](http://www.ncbi.nlm.nih.gov/pubmed/?term=Prunet%20C%5BAuthor%5D&cauthor=true&cauthor_uid=24034718), [Khoshnood B](http://www.ncbi.nlm.nih.gov/pubmed/?term=Khoshnood%20B%5BAuthor%5D&cauthor=true&cauthor_uid=24034718), [Blondel B](http://www.ncbi.nlm.nih.gov/pubmed/?term=Blondel%20B%5BAuthor%5D&cauthor=true&cauthor_uid=24034718).

[Author information](http://www.ncbi.nlm.nih.gov/pubmed/?term=Maternal+and+health+care+determinants+of+preconceptional+use+of+folic+acid+supplementation+in+France%3A+results+from+the+2010+National+Perinatal+Survey)

Abstract

OBJECTIVES:

To estimate the national prevalence and analyse the factors associated with preconceptional folic acid supplementation, including maternal sociodemographic characteristics, region of residence, birth control use and chronic diseases requiring medical care before conception.

DESIGN:

Cross-sectional population-based study.

SETTING:

All maternity units in France.

POPULATION:

A nationally representative sample of women giving birth in 2010 (n = 12,646).

METHODS:

Data came from mothers' interviews 2-3 days after delivery. Statistical analyses included multivariable logistic regressions.

MAIN OUTCOME MEASURE:

Folic acid supplementation starting at least 1 month before conception.

RESULTS:

14.8% (95% confidence interval [95% CI] 14.2-15.4) of women used folic acid before pregnancy; this percentage varied from 10.4% to 18.7% across regions. Supplementation was more frequent in primiparae, French citizens, women with higher educational levels and those needing medical monitoring or treatment before conception. Women who stopped contraception to become pregnant (75% of our population) usedfolic acid more often (intrauterine device or implant: 19%, pill: 17%, other methods which did not need medical monitoring: 17%) than other women (7%). The adjusted odds ratios were 3.3 (95% CI 2.6-4.3) for intrauterine device and implant, 2.2 (95% CI 1.8-2.6) for pill and 1.9 (95% CI 1.5-2.4) for other methods, compared with women who did not use birth control.

CONCLUSION:

The absence of preconceptional folic acid supplementation for most women, even those needing consultations with healthcare professionals before pregnancy, shows that campaigns to promote folic acid supplementation should address not only women but also healthcare professionals involved in birth control and obstetric care before pregnancy.

© 2013 Royal College of Obstetricians and Gynaecologists.

KEYWORDS:

Chronic diseases; fertility treat

Exclusion reasons: No autism data

10. [Hum Reprod Update.](http://www.ncbi.nlm.nih.gov/pubmed/?term=The+periconceptional+period%2C+reproduction+and+long-term+health+of+offspring%3A+the++importance+of+one-carbon+metabolism.) 2013 Nov-Dec;19(6):640-55. doi: 10.1093/humupd/dmt041. Epub 2013 Aug 19.

The periconceptional period, reproduction and long-term health of offspring: the importance of one-carbonmetabolism.

[Steegers-Theunissen RP](http://www.ncbi.nlm.nih.gov/pubmed/?term=Steegers-Theunissen%20RP%5BAuthor%5D&cauthor=true&cauthor_uid=23959022)1, [Twigt J](http://www.ncbi.nlm.nih.gov/pubmed/?term=Twigt%20J%5BAuthor%5D&cauthor=true&cauthor_uid=23959022), [Pestinger V](http://www.ncbi.nlm.nih.gov/pubmed/?term=Pestinger%20V%5BAuthor%5D&cauthor=true&cauthor_uid=23959022), [Sinclair KD](http://www.ncbi.nlm.nih.gov/pubmed/?term=Sinclair%20KD%5BAuthor%5D&cauthor=true&cauthor_uid=23959022).

[Author information](http://www.ncbi.nlm.nih.gov/pubmed/?term=The+periconceptional+period%2C+reproduction+and+long-term+health+of+offspring%3A+the++importance+of+one-carbon+metabolism.)

Abstract

BACKGROUND Most reproductive failures originate during the periconceptional period and are influenced by the age and the lifestyle of parents-to-be. We advance the hypothesis that these failures can arise as a partial consequence of derangements to one-carbon (1-C)metabolism (i.e. metabolic pathways that utilize substrates/cofactors such as methionine, vitamin B12, folate). 1-C metabolic pathways drive the synthesis of proteins, biogenic amines and lipids required for early growth, together with the synthesis and methylation of DNA and histones essential for the regulation of gene expression. We review how deficiencies in periconceptional 1-C metabolism affect fertility and development together with underlying mechanisms derived from animal studies. METHODS A literature search was performed using PubMed and bibliographies of all relevant original research articles and reviews. RESULTS We define 'periconception' as a 5-6-month period in women embracing oocyte growth, fertilization, conceptus formation and development to Week 10 of gestation (coinciding with the closure of the secondary palate in the embryo). During this period significant epigenetic modifications to chromatin occur that correspond with normal development. Subtle variations in 1-C metabolism genes and deficiencies in 1-C substrates/cofactors together with poor lifestyle, such as smoking and alcohol consumption, disturb 1-C metabolism and contribute to subfertility and early miscarriage and compromise offspring health. Procedures used in assisted reproductioncan also disturb these metabolic pathways and contribute to poor pregnancy outcomes. CONCLUSIONS Evidence presented indicates that parental nutrition and other lifestyle factors during the periconceptional period can affect reproductive performance via 1-C metabolic pathways. This knowledge provides opportunities for treatment and prevention of reproductive failures and future non-communicable diseases.

KEYWORDS:

B vitamins; epigenetics; fertility; folate; preconception care

Exclusions reason: Review

11. [Paediatr Perinat Epidemiol.](http://www.ncbi.nlm.nih.gov/pubmed/?term=Analysis+of+self-selection+bias+in+a+population-based+cohort+study+of+autism+spectrum+disorders.) 2013 Nov;27(6):553-63.

Analysis of self-selection bias in a population-based cohort study of autism spectrum disorders.

[Nilsen RM](http://www.ncbi.nlm.nih.gov/pubmed/?term=Nilsen%20RM%5BAuthor%5D&cauthor=true&cauthor_uid=23919580), [Surén P](http://www.ncbi.nlm.nih.gov/pubmed/?term=Sur%C3%A9n%20P%5BAuthor%5D&cauthor=true&cauthor_uid=23919580), [Gunnes N](http://www.ncbi.nlm.nih.gov/pubmed/?term=Gunnes%20N%5BAuthor%5D&cauthor=true&cauthor_uid=23919580), [Alsaker ER](http://www.ncbi.nlm.nih.gov/pubmed/?term=Alsaker%20ER%5BAuthor%5D&cauthor=true&cauthor_uid=23919580), [Bresnahan M](http://www.ncbi.nlm.nih.gov/pubmed/?term=Bresnahan%20M%5BAuthor%5D&cauthor=true&cauthor_uid=23919580), [Hirtz D](http://www.ncbi.nlm.nih.gov/pubmed/?term=Hirtz%20D%5BAuthor%5D&cauthor=true&cauthor_uid=23919580), [Hornig M](http://www.ncbi.nlm.nih.gov/pubmed/?term=Hornig%20M%5BAuthor%5D&cauthor=true&cauthor_uid=23919580), [Lie KK](http://www.ncbi.nlm.nih.gov/pubmed/?term=Lie%20KK%5BAuthor%5D&cauthor=true&cauthor_uid=23919580), [Lipkin WI](http://www.ncbi.nlm.nih.gov/pubmed/?term=Lipkin%20WI%5BAuthor%5D&cauthor=true&cauthor_uid=23919580), [Reichborn-Kjennerud T](http://www.ncbi.nlm.nih.gov/pubmed/?term=Reichborn-Kjennerud%20T%5BAuthor%5D&cauthor=true&cauthor_uid=23919580), [Roth C](http://www.ncbi.nlm.nih.gov/pubmed/?term=Roth%20C%5BAuthor%5D&cauthor=true&cauthor_uid=23919580), [Schjølberg S](http://www.ncbi.nlm.nih.gov/pubmed/?term=Schj%C3%B8lberg%20S%5BAuthor%5D&cauthor=true&cauthor_uid=23919580), [Smith GD](http://www.ncbi.nlm.nih.gov/pubmed/?term=Smith%20GD%5BAuthor%5D&cauthor=true&cauthor_uid=23919580),[Susser E](http://www.ncbi.nlm.nih.gov/pubmed/?term=Susser%20E%5BAuthor%5D&cauthor=true&cauthor_uid=23919580), [Vollset SE](http://www.ncbi.nlm.nih.gov/pubmed/?term=Vollset%20SE%5BAuthor%5D&cauthor=true&cauthor_uid=23919580), [Øyen AS](http://www.ncbi.nlm.nih.gov/pubmed/?term=%C3%98yen%20AS%5BAuthor%5D&cauthor=true&cauthor_uid=23919580), [Magnus P](http://www.ncbi.nlm.nih.gov/pubmed/?term=Magnus%20P%5BAuthor%5D&cauthor=true&cauthor_uid=23919580), [Stoltenberg C](http://www.ncbi.nlm.nih.gov/pubmed/?term=Stoltenberg%20C%5BAuthor%5D&cauthor=true&cauthor_uid=23919580).

Abstract

BACKGROUND:

This study examined potential self-selection bias in a large pregnancy cohort by comparing exposure-outcome associations from the cohort to similar associations obtained from nationwide registry data. The outcome under study was specialist-confirmed diagnosis ofautism spectrum disorders (ASDs).

METHODS:

The cohort sample (n = 89 836) was derived from the population-based prospective Norwegian Mother and Child Cohort Study and its substudy of ASDs, the Autism Birth Cohort (ABC) study. The nationwide registry data were derived from the Medical Birth Registry of Norway (n = 507 856). The children were born in 1999–2007, and seven prenatal and perinatal exposures were selected for analyses.

RESULTS:

ASDs were reported for 234 (0.26%) children in the cohort and 2072 (0.41%) in the nationwide population. Compared with the nationwide population, the cohort had an under-representation of the youngest women (<25 years), those who had single status, mothers who smoked during pregnancy, and non-users of prenatal folic acid supplements. The ratios of the adjusted odds ratios (ORs) in the cohort over the adjusted ORs in the nationwide population were as follows; primipara pregnancy: 1.39/1.22, prenatal folic acid use: 0.85/0.86, prenatal smoking: 1.20/1.17, preterm birth (<37 weeks): 1.48/1.42, low birthweight (<2500 g): 1.60/1.58, male sex: 4.39/4.59 (unadjusted only); and caesarean section history: 1.03/1.04.

CONCLUSIONS:

Associations estimated between ASDs and perinatal and prenatal exposures in the cohort are close to those estimated in the nationwide population. Self-selection does not appear to compromise validity of exposure-outcome associations in the ABC study.

Exclusion reasons: No folic acid data

12. [Epilepsia.](http://www.ncbi.nlm.nih.gov/pubmed/23865818) 2013 Aug;54(8):1462-72. doi: 10.1111/epi.12226. Epub 2013 Jul 19.

Exposure to antiepileptic drugs in utero and child development: a prospective population-based study.

[Veiby G](http://www.ncbi.nlm.nih.gov/pubmed/?term=Veiby%20G%5BAuthor%5D&cauthor=true&cauthor_uid=23865818)1, [Daltveit AK](http://www.ncbi.nlm.nih.gov/pubmed/?term=Daltveit%20AK%5BAuthor%5D&cauthor=true&cauthor_uid=23865818), [Schjølberg S](http://www.ncbi.nlm.nih.gov/pubmed/?term=Schj%C3%B8lberg%20S%5BAuthor%5D&cauthor=true&cauthor_uid=23865818), [Stoltenberg C](http://www.ncbi.nlm.nih.gov/pubmed/?term=Stoltenberg%20C%5BAuthor%5D&cauthor=true&cauthor_uid=23865818), [Øyen AS](http://www.ncbi.nlm.nih.gov/pubmed/?term=%C3%98yen%20AS%5BAuthor%5D&cauthor=true&cauthor_uid=23865818), [Vollset SE](http://www.ncbi.nlm.nih.gov/pubmed/?term=Vollset%20SE%5BAuthor%5D&cauthor=true&cauthor_uid=23865818), [Engelsen BA](http://www.ncbi.nlm.nih.gov/pubmed/?term=Engelsen%20BA%5BAuthor%5D&cauthor=true&cauthor_uid=23865818), [Gilhus NE](http://www.ncbi.nlm.nih.gov/pubmed/?term=Gilhus%20NE%5BAuthor%5D&cauthor=true&cauthor_uid=23865818).

[Author information](http://www.ncbi.nlm.nih.gov/pubmed/23865818)

Abstract

PURPOSE:

Antiepileptic drugs may cause congenital malformations. Less is known about the effect on development in infancy and childhood. The aim of this study was to examine whether exposure to antiepileptic drugs during pregnancy has an effect on early child development.

METHODS:

From mid-1999 through December 2008, children of mothers recruited at 13-17 weeks of pregnancy were studied in the ongoingprospective Norwegian Mother and Child Cohort Study. Information on birth outcomes were obtained from the Medical Birth Registry (108,264 children), and mothers reported on their child's motor development, language, social skills, and autistic traits using items from standardized screening tools at 18 months (61,351 children) and 36 months (44,147 children) of age. The relative risk of adverse outcomes in children according to maternal or paternal epilepsy with and without prenatal exposure to antiepileptic drugs was estimated as odds ratios (ORs), using logistic regression with adjustment for maternal age, parity, education, smoking, depression/anxiety, folate supplementation, and child congenital malformation or low birth weight.

KEY FINDINGS:

A total of 333 children were exposed to antiepileptic drugs in utero. At 18 months, the exposed children had increased risk of abnormal scores for gross motor skills (7.1% vs. 2.9%; OR 2.0, 95% confidence interval [CI] 1.1-3.7) and autistic traits (3.5% vs. 0.9%; OR 2.7, CI 1.1-6.7) compared to children of parents without epilepsy. At 36 months, the exposed children had increased risk of abnormal score for gross motor skills (7.5% vs. 3.3%; OR 2.2, CI 1.1-4.2), sentence skills (11.2% vs. 4.8%; OR 2.1, CI 1.2-3.6), and autistic traits (6.0% vs. 1.5%; OR 3.4, CI 1.6-7.0). The drug-exposed children also had increased risk of congenital malformations (6.1% vs. 2.9%; OR 2.1, CI 1.4-3.4), but exclusion of congenital malformations did not affect the risk of adverse development. Children born to women with epilepsy who did not use antiepileptic drugs had no increased risks. Children of fathers with epilepsy generally scored within the normal range.

SIGNIFICANCE:

Exposure to antiepileptic drugs during pregnancy is associated with adverse development at 18 and 36 months of age, measured as low scores within key developmental domains rated by mothers. Exposures to valproate, lamotrigine, carbamazepine, or multipleantiepileptic drugs were associated with adverse outcome within different developmental domains.

Wiley Periodicals, Inc. © 2013 International League Against Epilepsy.

KEYWORDS:

Epilepsy; MoBa study; Pregnancy; Teratogenicity

Exclusion reasons: No folic acid data

13. J Midwifery Womens Health. 2013 Jul-Aug;58(4):471-2. doi: 10.1111/jmwh.12081_1.

Epub 2013 Jul 9.

Folic acid supplementation before and in early pregnancy may decrease risk for

autism.

Faucher MA.

Exclusion reasons: Commentary

14. J Pediatr. 2013 Jul;163(1):303-4. doi: 10.1016/j.jpeds.2013.04.060.

Maternal prenatal folic acid supplementation is associated with a reduction in

development of autistic disorder.

Berry RJ(1).

Exclusion reasons: Commentary

15. JAMA. 2013 Jun 5;309(21):2208. doi: 10.1001/jama.2013.4876.

Maternal folic acid supplementation and risk of autism.

Vahabzadeh A, McDougle CJ.

Exclusion reasons: Commentary

16. Evid Based Med. 2013 Dec;18(6):e53. doi: 10.1136/eb-2013-101311. Epub 2013 May 1.

Maternal folic acid supplements associated with reduced autism risk in the child.

Schmidt RJ(1).

Exclusion reasons: Commentary

17. JAMA. 2013 Feb 13;309(6):611-3. doi: 10.1001/jama.2013.198.

Periconceptional folic acid and risk of autism spectrum disorders.

Berry RJ, Crider KS, Yeargin-Allsopp M.

Exclusion reasons: Commentary

18. [Lancet Neurol.](http://www.ncbi.nlm.nih.gov/pubmed/?term=Fetal+antiepileptic+drug+exposure+and+cognitive+outcomes+at+age+6+years+(NEAD+study)%3A+a+prospective+observational+study.) 2013 Mar;12(3):244-52. doi: 10.1016/S1474-4422(12)70323-X. Epub 2013 Jan 23.

Fetal antiepileptic drug exposure and cognitive outcomes at age 6 years (NEAD study): a prospectiveobservational study.

[Meador KJ](http://www.ncbi.nlm.nih.gov/pubmed/?term=Meador%20KJ%5BAuthor%5D&cauthor=true&cauthor_uid=23352199), [Baker GA](http://www.ncbi.nlm.nih.gov/pubmed/?term=Baker%20GA%5BAuthor%5D&cauthor=true&cauthor_uid=23352199), [Browning N](http://www.ncbi.nlm.nih.gov/pubmed/?term=Browning%20N%5BAuthor%5D&cauthor=true&cauthor_uid=23352199), [Cohen MJ](http://www.ncbi.nlm.nih.gov/pubmed/?term=Cohen%20MJ%5BAuthor%5D&cauthor=true&cauthor_uid=23352199), [Bromley RL](http://www.ncbi.nlm.nih.gov/pubmed/?term=Bromley%20RL%5BAuthor%5D&cauthor=true&cauthor_uid=23352199), [Clayton-Smith J](http://www.ncbi.nlm.nih.gov/pubmed/?term=Clayton-Smith%20J%5BAuthor%5D&cauthor=true&cauthor_uid=23352199), [Kalayjian LA](http://www.ncbi.nlm.nih.gov/pubmed/?term=Kalayjian%20LA%5BAuthor%5D&cauthor=true&cauthor_uid=23352199), [Kanner A](http://www.ncbi.nlm.nih.gov/pubmed/?term=Kanner%20A%5BAuthor%5D&cauthor=true&cauthor_uid=23352199), [Liporace JD](http://www.ncbi.nlm.nih.gov/pubmed/?term=Liporace%20JD%5BAuthor%5D&cauthor=true&cauthor_uid=23352199), [Pennell PB](http://www.ncbi.nlm.nih.gov/pubmed/?term=Pennell%20PB%5BAuthor%5D&cauthor=true&cauthor_uid=23352199), [Privitera M](http://www.ncbi.nlm.nih.gov/pubmed/?term=Privitera%20M%5BAuthor%5D&cauthor=true&cauthor_uid=23352199), [Loring DW](http://www.ncbi.nlm.nih.gov/pubmed/?term=Loring%20DW%5BAuthor%5D&cauthor=true&cauthor_uid=23352199); [NEADStudy Group](http://www.ncbi.nlm.nih.gov/pubmed/?term=NEAD%20Study%20Group%5BCorporate%20Author%5D).

[Collaborators (98)](http://www.ncbi.nlm.nih.gov/pubmed/?term=Fetal+antiepileptic+drug+exposure+and+cognitive+outcomes+at+age+6+years+(NEAD+study)%3A+a+prospective+observational+study.)

Abstract

BACKGROUND:

Many women of childbearing potential take antiepileptic drugs, but the cognitive effects of fetal exposure are uncertain. We aimed to assess effects of commonly used antiepileptic drugs on cognitive outcomes in children up to 6 years of age.

METHODS:

In this prospective, observational, assessor-masked, multicentre study, we enrolled pregnant women with epilepsy on antiepilepticdrug monotherapy (carbamazepine, lamotrigine, phenytoin, or valproate) between October, 1999, and February, 2004, at 25 epilepsy centres in the UK and the USA. Our primary outcome was intelligence quotient (IQ) at 6 years of age (age-6 IQ) in all children, assessed with linear regression adjusted for maternal IQ, antiepileptic drug type, standardised dose, gestational birth age, and use of periconceptional folate. We also assessed multiple cognitive domains and compared findings with outcomes at younger ages. This study is registered with ClinicalTrials.gov, number [NCT00021866](http://clinicaltrials.gov/show/NCT00021866).

FINDINGS:

We included 305 mothers and 311 children (six twin pairs) in the primary analysis. 224 children completed 6 years of follow-up (6-year-completer sample). Multivariate analysis of all children showed that age-6 IQ was lower after exposure to valproate (mean 97, 95% CI 94-101) than to carbamazepine (105, 102-108; p=0·0015), lamotrigine (108, 105-110; p=0·0003), or phenytoin (108, 104-112; p=0·0006). Children exposed to valproate did poorly on measures of verbal and memory abilities compared with those exposed to the other antiepileptic drugs and on non-verbal and executive functions compared with lamotrigine (but not carbamazepine or phenytoin). High doses of valproate were negatively associated with IQ (r=-0·56, p<0·0001), verbal ability (r=-0·40, p=0·0045), non-verbal ability (r=-0·42, p=0·0028), memory (r=-0·30, p=0·0434), and executive function (r=-0·42, p=0·0004), but other antiepileptic drugs were not. Age-6 IQ correlated with IQs at younger ages, and IQ improved with age for infants exposed to any antiepileptic drug. Compared with a normative sample (173 [93%] of 187 children), right-handedness was less frequent in children in our study overall (185 [86%] of 215; p=0·0404) and in the lamotrigine (59 [83%] of 71; p=0·0287) and valproate (38 [79%] of 40; p=0·0089) groups. Verbal abilities were worse than non-verbal abilities in children in our study overall and in the lamotrigine and valproate groups. Mean IQs were higher in children exposed to periconceptional folate (108, 95% CI 106-111) than they were in unexposed children (101, 98-104; p=0·0009).

INTERPRETATION:

Fetal valproate exposure has dose-dependent associations with reduced cognitive abilities across a range of domains at 6years of age. Reduced right-handedness and verbal (vs non-verbal) abilities might be attributable to changes in cerebral lateralisation induced byexposure to antiepileptic drugs. The positive association of periconceptional folate with IQ is consistent with other recent studies.

Exclusion reasons: No folic acid data

19. [Nutrients.](http://www.ncbi.nlm.nih.gov/pubmed/?term=Benefits+of+docosahexaenoic+acid%2C+folic+acid%2C+vitamin+D+and+iodine+on+foetal+and++infant+brain+development+and+function+following+maternal+supplementation+during+pregnancy+and+lactation.) 2012 Jul;4(7):799-840. doi: 10.3390/nu4070799. Epub 2012 Jul 24.

Benefits of docosahexaenoic acid, folic acid, vitamin D and iodine on foetal and infant brain development andfunction following maternal supplementation during pregnancy and lactation.

[Morse NL](http://www.ncbi.nlm.nih.gov/pubmed/?term=Morse%20NL%5BAuthor%5D&cauthor=true&cauthor_uid=22852064)1.

[Author information](http://www.ncbi.nlm.nih.gov/pubmed/?term=Benefits+of+docosahexaenoic+acid%2C+folic+acid%2C+vitamin+D+and+iodine+on+foetal+and++infant+brain+development+and+function+following+maternal+supplementation+during+pregnancy+and+lactation.)

Abstract

Scientific literature is increasingly reporting on dietary deficiencies in many populations of some nutrients critical for foetal and infant braindevelopment and function.

PURPOSE:

To highlight the potential benefits of maternal supplementation with docosahexaenoic acid (DHA) and other important complimentary nutrients, including vitamin D, folic acid and iodine during pregnancy and/or breast feeding for foetal and/or infant braindevelopment and/or function.

METHODS:

English language systematic reviews, meta-analyses, randomised controlled trials, cohort studies, cross-sectional and case-control studies were obtained through searches on MEDLINE and the Cochrane Register of Controlled Trials from January 2000 through to February 2012 and reference lists of retrieved articles. Reports were selected if they included benefits and harms of maternal supplementation of DHA, vitaminD, folic acid or iodine supplementation during pregnancy and/or lactation.

RESULTS:

Maternal DHA intake during pregnancy and/or lactation can prolong high risk pregnancies, increase birth weight, head circumference and birth length, and can enhance visual acuity, hand and eye co-ordination, attention, problem solving and information processing. Vitamin Dhelps maintain pregnancy and promotes normal skeletal and brain development. Folic acid is necessary for normal foetal spine, brain and skulldevelopment. Iodine is essential for thyroid hormone production necessary for normal brain and nervous system development during gestation that impacts childhood function.

CONCLUSION:

Maternal supplementation within recommended safe intakes in populations with dietary deficiencies may prevent many brainand central nervous system malfunctions and even enhance brain development and function in their offspring.

KEYWORDS:

DHA; brain development; brain function; docosahexaenoic acid; eye function; foetal development; folic acid; infant development;iodine; vitamin D

Exclusion reasons: Review

20. [Crit Rev Food Sci Nutr.](http://www.ncbi.nlm.nih.gov/pubmed/22823344) 2012;52(11):959-64. doi: 10.1080/10408398.2010.515042.

Effects of prenatal and/or postnatal (maternal and/or child) folic acid supplementation on the mentalperformance of children.

[Skórka A](http://www.ncbi.nlm.nih.gov/pubmed/?term=Sk%C3%B3rka%20A%5BAuthor%5D&cauthor=true&cauthor_uid=22823344)1, [Gieruszczak-Białek D](http://www.ncbi.nlm.nih.gov/pubmed/?term=Gieruszczak-Bia%C5%82ek%20D%5BAuthor%5D&cauthor=true&cauthor_uid=22823344), [Pieścik M](http://www.ncbi.nlm.nih.gov/pubmed/?term=Pie%C5%9Bcik%20M%5BAuthor%5D&cauthor=true&cauthor_uid=22823344), [Szajewska H](http://www.ncbi.nlm.nih.gov/pubmed/?term=Szajewska%20H%5BAuthor%5D&cauthor=true&cauthor_uid=22823344).

[Author information](http://www.ncbi.nlm.nih.gov/pubmed/22823344)

Abstract

It has been suggested that a deficiency in folic acid during early, critical central nervous system development may result in persistent cognitive and behavioral effects. The purpose of this systematic review was to evaluate evidence regarding whether folic acid supplementation during pregnancy and early life influences mental performance outcomes in children. The following electronic databases were searched through December 2009 for studies relevant to mental performance and folic acid: MEDLINE, EMBASE and The Cochrane Library; additional references were obtained from reviewed articles. Only randomized controlled trials (RCTs) were included. Of 8 RCTs identified, only 2 met the inclusion criteria. Both studies involved periconceptional, multivitamin-containing, folic acid supplementation. Evidence from these 2 RCTs suggests that such supplementation does not affect the postnatal mental development of infants at a mean age of 11 mo, the developmental quotient (DQ) at 2 y of age, or the intelligence quotient (IQ) and Goodenough man drawing test quotient (DrQ) at 6 y of age. We conclude that the use of multivitamin-containing folic acid supplementation during pregnancy is associated with no benefit to the mental performance of children. These findings should be interpreted with caution due to the very limited number of studies included in this systemic review.

Exclusion reasons: Review

21. [Environ Res.](http://www.ncbi.nlm.nih.gov/pubmed/?term=Longitudinal+association+between+early+life+socio-environmental+factors+and+attention+function+at+the+age+11+years.) 2012 Aug;117:54-9. doi: 10.1016/j.envres.2012.04.007. Epub 2012 May 18.

Longitudinal association between early life socio-environmental factors and attention function at the age 11years.

[Forns J](http://www.ncbi.nlm.nih.gov/pubmed/?term=Forns%20J%5BAuthor%5D&cauthor=true&cauthor_uid=22608140)1, [Torrent M](http://www.ncbi.nlm.nih.gov/pubmed/?term=Torrent%20M%5BAuthor%5D&cauthor=true&cauthor_uid=22608140), [Garcia-Esteban R](http://www.ncbi.nlm.nih.gov/pubmed/?term=Garcia-Esteban%20R%5BAuthor%5D&cauthor=true&cauthor_uid=22608140), [Cáceres A](http://www.ncbi.nlm.nih.gov/pubmed/?term=C%C3%A1ceres%20A%5BAuthor%5D&cauthor=true&cauthor_uid=22608140), [Pilar Gomila M](http://www.ncbi.nlm.nih.gov/pubmed/?term=Pilar%20Gomila%20M%5BAuthor%5D&cauthor=true&cauthor_uid=22608140), [Martinez D](http://www.ncbi.nlm.nih.gov/pubmed/?term=Martinez%20D%5BAuthor%5D&cauthor=true&cauthor_uid=22608140), [Morales E](http://www.ncbi.nlm.nih.gov/pubmed/?term=Morales%20E%5BAuthor%5D&cauthor=true&cauthor_uid=22608140), [Julvez J](http://www.ncbi.nlm.nih.gov/pubmed/?term=Julvez%20J%5BAuthor%5D&cauthor=true&cauthor_uid=22608140), [Grimalt JO](http://www.ncbi.nlm.nih.gov/pubmed/?term=Grimalt%20JO%5BAuthor%5D&cauthor=true&cauthor_uid=22608140), [Sunyer J](http://www.ncbi.nlm.nih.gov/pubmed/?term=Sunyer%20J%5BAuthor%5D&cauthor=true&cauthor_uid=22608140).

[Author information](http://www.ncbi.nlm.nih.gov/pubmed/?term=Longitudinal+association+between+early+life+socio-environmental+factors+and+attention+function+at+the+age+11+years.)

Abstract

Prenatal and early-life exposures can affect the course of children's neuropsychological development well into pre-adolescence, given the vulnerability of the developing brain. However, it is unknown which socio-environmental factors at early childhood can influence specific cognitive processes like attention at a later age. In this study, we aim to determine social and environmental exposures in early childhood that may be associated with attention function of 11-year-olds. We measured attention function using the continuous performance test-II (CPT-II) on 393 11-year old children from the Menorca's birth-cohort within the INMA-project (Spain), and pre-selected a list of socio-environmentalobservations taken when they were up to 4 years of age. We found that earlier socio-environmental characteristics, such as parental social class, educational level and maternal mental health are associated with later inattentive and impulsive symptomatology through a higher rate of omission and commission errors. In addition, omission errors were higher in children with atopy and lower in those whose mothers took dietary supplementation with folic acid and vitamins during pregnancy. Breastfeeding played a protective role against commission errors, while higher DDE and PCBs levels at age 4 were associated with slow speed response. Our findings suggest that a number of life socio-environmental factorsduring prenatal life and early childhood, such as socio-demographic characteristics, breastfeeding, maternal nutritional supplementation with folic acid and vitamins and exposure to some organochlorine compounds may influence inattentive and hyperactive/impulsive symptomatology during pre-adolescence.

Exclusion reasons: No autism data.

22. [J Dev Orig Health Dis.](http://www.ncbi.nlm.nih.gov/pubmed/?term=Vitamin+B12+and+folate+during+pregnancy+and+offspring+motor%2C+mental+and+social+development+at+2+years+of+age) 2012 Apr;3(2):123-30. doi: 10.1017/S2040174411000778.

Vitamin B12 and folate during pregnancy and offspring motor, mental and social development at 2 years ofage.

[Bhate VK](http://www.ncbi.nlm.nih.gov/pubmed/?term=Bhate%20VK%5BAuthor%5D&cauthor=true&cauthor_uid=25101923)1, [Joshi SM](http://www.ncbi.nlm.nih.gov/pubmed/?term=Joshi%20SM%5BAuthor%5D&cauthor=true&cauthor_uid=25101923)1, [Ladkat RS](http://www.ncbi.nlm.nih.gov/pubmed/?term=Ladkat%20RS%5BAuthor%5D&cauthor=true&cauthor_uid=25101923)1, [Deshmukh US](http://www.ncbi.nlm.nih.gov/pubmed/?term=Deshmukh%20US%5BAuthor%5D&cauthor=true&cauthor_uid=25101923)1, [Lubree HG](http://www.ncbi.nlm.nih.gov/pubmed/?term=Lubree%20HG%5BAuthor%5D&cauthor=true&cauthor_uid=25101923)1, [Katre PA](http://www.ncbi.nlm.nih.gov/pubmed/?term=Katre%20PA%5BAuthor%5D&cauthor=true&cauthor_uid=25101923)2, [Bhat DS](http://www.ncbi.nlm.nih.gov/pubmed/?term=Bhat%20DS%5BAuthor%5D&cauthor=true&cauthor_uid=25101923)1, [Rush EC](http://www.ncbi.nlm.nih.gov/pubmed/?term=Rush%20EC%5BAuthor%5D&cauthor=true&cauthor_uid=25101923)3, [Yajnik CS](http://www.ncbi.nlm.nih.gov/pubmed/?term=Yajnik%20CS%5BAuthor%5D&cauthor=true&cauthor_uid=25101923)1.

[Author information](http://www.ncbi.nlm.nih.gov/pubmed/?term=Vitamin+B12+and+folate+during+pregnancy+and+offspring+motor%2C+mental+and+social+development+at+2+years+of+age)

Abstract

Insufficiency of vitamin B12 (B12) and folate during pregnancy can result in low concentrations in the fetus and have adverse effects on braindevelopment. We investigated the relationship between maternal B12 and folate nutrition during pregnancy and offspring motor, mental andsocial development at two years of age (2 y). Mothers (n = 123) and their offspring (62 girls, 61 boys) from rural and middle-class urban communities in and around Pune city were followed through pregnancy up to 2 y. Maternal B12 and folate concentrations were measured at 28 and 34 weeks of gestation. At 2 y, the Developmental Assessment Scale for Indian Infants was used to determine motor and mentaldevelopmental quotients and the Vineland Social Maturity Scale for the social developmental quotient. Overall, 62% of the mothers had low B12levels (<150 pmol/l) and one mother was folate deficient during pregnancy. Maternal B12 at 28 and 34 weeks of gestation was associated withoffspring B12 at 2 y (r = 0.29, r = 0.32, P < 0.001), but folate was not associated with offspring folate. At 2 y, motor development was associated with maternal folate at 28 and 34 weeks of gestation. Mental and social development quotients were associated positively with head circumference and negatively with birth weight. In addition, pregnancy B12 and folate were positively associated with mental and socialdevelopment quotients. Maternal B12 and folate during intrauterine life may favorably influence brain development and function. Pregnancyprovides a window of opportunity to enhance fetal psychomotor (motor and mental) development.

Exclusion reasons: No autism data.

23. [Am J Clin Nutr.](http://www.ncbi.nlm.nih.gov/pubmed/?term=Impact+of+prenatal+multiple+micronutrients+on+survival+and+growth+during+infancy%3A+a+randomized+controlled+trial.) 2012 Apr;95(4):916-24. doi: 10.3945/ajcn.111.029033. Epub 2012 Feb 29.

Impact of prenatal multiple micronutrients on survival and growth during infancy: a randomized controlledtrial.

[Roberfroid D](http://www.ncbi.nlm.nih.gov/pubmed/?term=Roberfroid%20D%5BAuthor%5D&cauthor=true&cauthor_uid=22378724)1, [Huybregts L](http://www.ncbi.nlm.nih.gov/pubmed/?term=Huybregts%20L%5BAuthor%5D&cauthor=true&cauthor_uid=22378724), [Lanou H](http://www.ncbi.nlm.nih.gov/pubmed/?term=Lanou%20H%5BAuthor%5D&cauthor=true&cauthor_uid=22378724), [Ouedraogo L](http://www.ncbi.nlm.nih.gov/pubmed/?term=Ouedraogo%20L%5BAuthor%5D&cauthor=true&cauthor_uid=22378724), [Henry MC](http://www.ncbi.nlm.nih.gov/pubmed/?term=Henry%20MC%5BAuthor%5D&cauthor=true&cauthor_uid=22378724), [Meda N](http://www.ncbi.nlm.nih.gov/pubmed/?term=Meda%20N%5BAuthor%5D&cauthor=true&cauthor_uid=22378724), [Kolsteren P](http://www.ncbi.nlm.nih.gov/pubmed/?term=Kolsteren%20P%5BAuthor%5D&cauthor=true&cauthor_uid=22378724); [MISAME study group](http://www.ncbi.nlm.nih.gov/pubmed/?term=MISAME%20study%20group%5BCorporate%20Author%5D).

[Collaborators (8)](http://www.ncbi.nlm.nih.gov/pubmed/?term=Impact+of+prenatal+multiple+micronutrients+on+survival+and+growth+during+infancy%3A+a+randomized+controlled+trial.)

[Author information](http://www.ncbi.nlm.nih.gov/pubmed/?term=Impact+of+prenatal+multiple+micronutrients+on+survival+and+growth+during+infancy%3A+a+randomized+controlled+trial.)

Abstract

BACKGROUND:

Although prenatal multiple micronutrients can improve fetal growth, their benefit on postnatal health remains uncertain.

OBJECTIVE:

We assessed the effect of the UNICEF/WHO/United Nations University multiple micronutrient supplement for pregnant and lactating women (UNIMMAP) compared with the usual iron and folic acid supplement (IFA) on survival, growth, and morbidity during infancy.

DESIGN:

In a double-blind, randomized trial, we followed 1294 singleton newborns whose mothers had prenatally received either the UNIMMAP or IFA. We assessed monthly anthropometric measures and health variables up to age 12 mo. Children were assessed again at a mean age of 30 mo. Mixed-effects models accounted for repeated measurements.

RESULTS:

The UNIMMAP resulted in a 27% (HR: 0.73; 95% CI: 0.60, 0.87; P = 0.002) reduction in the rate of stunting in 15,261 infant-months with a higher length-for-age z score of 0.13 (95% CI: 0.02, 0.24; P = 0.02) over the whole observation period. However, by age 30 mo, this difference was not observed. An effect of the UNIMMAP on weight-for-length (P-interaction = 0.004) and head circumference-for-age (P-interaction = 0.03) became apparent by the end of the first year of life. By the age of 30 mo, children from the UNIMMAP group had a higher weight-for-height z score of 0.20 (95% CI: 0.06, 0.34; P = 0.004). No difference in mortality or morbidity was identified in groups, except a 14% reduction in reported episodes of fever (95% CI: 1%, 28%; P = 0.04).

CONCLUSIONS:

Improved linear fetal growth with continuation into early life and enhanced postnatal growth were 2 mechanisms that mediated the effect of the prenatal UNIMMAP on infant nutritional status. Additional follow-up to assess long-term effects is warranted.

Exclusion reasons: No autism data.

24. [Nestle Nutr Inst Workshop Ser.](http://www.ncbi.nlm.nih.gov/pubmed/25825305) 2012;70:161-71. doi: 10.1159/000337684. Epub 2012 Aug 31.

Folate and vitamin B12: function and importance in cognitive development.

[Troen AM](http://www.ncbi.nlm.nih.gov/pubmed/?term=Troen%20AM%5BAuthor%5D&cauthor=true&cauthor_uid=25825305)1.

[Author information](http://www.ncbi.nlm.nih.gov/pubmed/25825305)

- 1Nutrition and Brain Health Laboratory, Institute of Biochemistry, Food Science and Nutrition, The Robert H. Smith Faculty of Agriculture, Food and Environment, The Hebrew University of Jerusalem, Rehovot, Israel.

Abstract

The importance of the B vitamins folate and vitamin B12 for healthy neurological development and function is unquestioned. Folate andvitamin B12 are required for biological methylation and DNA synthesis. Vitamin B12 also participates in the mitochondrial catabolism of odd-chain fatty acids and some amino acids. Inborn errors of their metabolism and severe nutritional deficiencies cause serious neurological and hematological pathology. Poor folate and vitamin B12 status short of clinical deficiency is associated with increased risk of cognitive impairment, depression, Alzheimer's disease and stroke among older adults and increased risk of neural tube defects among children born to mothers with lowfolate status. Folate supplementation and food fortification are known to reduce incident neural tube defects, and B vitamin supplementation may have cognitive benefit in older adults. Less is known about folate and vitamin B12 requirements for optimal brain development and long-termcognitive health in newborns, children and adolescents. While increasing suboptimal nutritional status has observed benefits, the long-term effects of high folate intake are uncertain. Several observations of unfavorable health indicators in children and adults exposed to high folic acid intake make it imperative to achieve a more precise definition of folate and B12 requirements for brain development and function.

Exclusion reasons: commentary

25. [Matern Child Nutr.](http://www.ncbi.nlm.nih.gov/pubmed/22023381) 2013 Apr;9(2):155-66. doi: 10.1111/j.1740-8709.2011.00364.x. Epub 2011 Oct 24.

Folate, vitamin B12, vitamin B6 and homocysteine: impact on pregnancy outcome.

[Furness D](http://www.ncbi.nlm.nih.gov/pubmed/?term=Furness%20D%5BAuthor%5D&cauthor=true&cauthor_uid=22023381)1, [Fenech M](http://www.ncbi.nlm.nih.gov/pubmed/?term=Fenech%20M%5BAuthor%5D&cauthor=true&cauthor_uid=22023381), [Dekker G](http://www.ncbi.nlm.nih.gov/pubmed/?term=Dekker%20G%5BAuthor%5D&cauthor=true&cauthor_uid=22023381), [Khong TY](http://www.ncbi.nlm.nih.gov/pubmed/?term=Khong%20TY%5BAuthor%5D&cauthor=true&cauthor_uid=22023381), [Roberts C](http://www.ncbi.nlm.nih.gov/pubmed/?term=Roberts%20C%5BAuthor%5D&cauthor=true&cauthor_uid=22023381), [Hague W](http://www.ncbi.nlm.nih.gov/pubmed/?term=Hague%20W%5BAuthor%5D&cauthor=true&cauthor_uid=22023381).

[Author information](http://www.ncbi.nlm.nih.gov/pubmed/22023381)

Abstract

Good clinical practice recommends folic acid supplementation 1 month prior to pregnancy and during the first trimester to prevent congenital malformations. However, high rates of fetal growth and development in later pregnancy may increase the demand for folate. Folate and vitaminsB12 and B6 are required for DNA synthesis and cell growth, and are involved in homocysteine metabolism. The primary aim of this study was to determine if maternal folate, vitamin B12, vitamin B6 and homocysteine concentrations at 18-20 weeks gestation are associated with subsequent adverse pregnancy outcomes, including pre-eclampsia and intrauterine growth restriction (IUGR). The secondary aim was to investigate maternal B vitamin concentrations with DNA damage markers in maternal lymphocytes. A prospective observational study was conducted at the Women's and Children's Hospital, Adelaide, South Australia. One hundred and thirty-seven subjects were identified prior to 20 weeks gestation as at high or low risk for subsequent adverse pregnancy outcome by senior obstetricians. Clinical status, dietary information, circulating micronutrients and genome damage biomarkers were assessed at 18-20 weeks gestation. Women who developed IUGR had reduced red blood cell (RBC) folate (P < 0.001) and increased plasma homocysteine concentrations (P < 0.001) compared with controls. Maternal DNA damage, represented by micronucleus frequency and nucleoplasmic bridges in lymphocytes, was positively correlated with homocysteine(r = 0.179, P = 0.038 and r = 0.171, P = 0.047, respectively). Multivariate regression analysis revealed RBC folate was a strong predictor of IUGR (P = 0.006). This study suggests that low maternal RBC folate and high homocysteine values in mid pregnancy are associated with subsequent reduced fetal growth.

Exclusion reasons: No autism data.

26. [Brain.](http://www.ncbi.nlm.nih.gov/pubmed/?term=Foetal+antiepileptic+drug+exposure+and+verbal+versus+non-verbal+abilities+at+three+years+of+age.) 2011 Feb;134(Pt 2):396-404. doi: 10.1093/brain/awq352. Epub 2011 Jan 11.

Foetal antiepileptic drug exposure and verbal versus non-verbal abilities at three years of age.

[Meador KJ](http://www.ncbi.nlm.nih.gov/pubmed/?term=Meador%20KJ%5BAuthor%5D&cauthor=true&cauthor_uid=21224309), [Baker GA](http://www.ncbi.nlm.nih.gov/pubmed/?term=Baker%20GA%5BAuthor%5D&cauthor=true&cauthor_uid=21224309), [Browning N](http://www.ncbi.nlm.nih.gov/pubmed/?term=Browning%20N%5BAuthor%5D&cauthor=true&cauthor_uid=21224309), [Cohen MJ](http://www.ncbi.nlm.nih.gov/pubmed/?term=Cohen%20MJ%5BAuthor%5D&cauthor=true&cauthor_uid=21224309), [Clayton-Smith J](http://www.ncbi.nlm.nih.gov/pubmed/?term=Clayton-Smith%20J%5BAuthor%5D&cauthor=true&cauthor_uid=21224309), [Kalayjian LA](http://www.ncbi.nlm.nih.gov/pubmed/?term=Kalayjian%20LA%5BAuthor%5D&cauthor=true&cauthor_uid=21224309), [Kanner A](http://www.ncbi.nlm.nih.gov/pubmed/?term=Kanner%20A%5BAuthor%5D&cauthor=true&cauthor_uid=21224309), [Liporace JD](http://www.ncbi.nlm.nih.gov/pubmed/?term=Liporace%20JD%5BAuthor%5D&cauthor=true&cauthor_uid=21224309), [Pennell PB](http://www.ncbi.nlm.nih.gov/pubmed/?term=Pennell%20PB%5BAuthor%5D&cauthor=true&cauthor_uid=21224309), [Privitera M](http://www.ncbi.nlm.nih.gov/pubmed/?term=Privitera%20M%5BAuthor%5D&cauthor=true&cauthor_uid=21224309), [Loring DW](http://www.ncbi.nlm.nih.gov/pubmed/?term=Loring%20DW%5BAuthor%5D&cauthor=true&cauthor_uid=21224309); [NEAD Study Group](http://www.ncbi.nlm.nih.gov/pubmed/?term=NEAD%20Study%20Group%5BCorporate%20Author%5D).

[Collaborators (100)](http://www.ncbi.nlm.nih.gov/pubmed/?term=Foetal+antiepileptic+drug+exposure+and+verbal+versus+non-verbal+abilities+at+three+years+of+age.)

Abstract

We previously reported that foetal valproate exposure impairs intelligence quotient. In this follow-up investigation, we examined dose-related effects of foetal antiepileptic drug exposure on verbal and non-verbal cognitive measures. This investigation is an ongoing prospective observational multi-centre study in the USA and UK, which has enrolled pregnant females with epilepsy on monotherapy from 1999 to 2004. The study seeks to determine if differential long-term neurodevelopmental effects exist across four commonly used drugs (carbamazepine, lamotrigine, phenytoin and valproate). This report compares verbal versus non-verbal cognitive outcomes in 216 children who completed testing at the age ofthree years. Verbal and non-verbal index scores were calculated from the Differential Ability Scales, Preschool Language Scale, Peabody Picture Vocabulary Test and Developmental Test of Visual-Motor Integration. Verbal abilities were lower than non-verbal in children exposed in utero to each drug. Preconceptional folate use was associated with higher verbal outcomes. Valproate was associated with poorer cognitive outcomes. Performance was negatively associated with valproate dose for both verbal and non-verbal domains and negatively associated with carbamazepine dose for verbal performance. No dose effects were seen for lamotrigine and phenytoin. Since foetal antiepileptic drug exposureis associated with lower verbal than non-verbal abilities, language may be particularly susceptible to foetal exposure. We hypothesize thatfoetal drug exposure may alter normal cerebral lateralization. Further, a dose-dependent relationship is present for both lower verbal and non-verbal abilities with valproate and for lower verbal abilities with carbamazepine. Preconceptional folate may improve cognitive outcomes. Additional research is needed to confirm these findings, extend the study to other drugs, define the risks associated with drug treatment for seizures in the neonates, and understand the underlying mechanisms.

Exclusion reasons: No folic acid data.

27. [Am J Obstet Gynecol.](http://www.ncbi.nlm.nih.gov/pubmed/?term=Maternal+depressive+symptoms%2C+serum+folate+status%2C+and+pregnancy+outcome%3A+results+of+the+Amsterdam+Born+Children+and+their+Development+study.) 2010 Dec;203(6):563.e1-7. doi: 10.1016/j.ajog.2010.07.017. Epub 2010 Sep 15.

# Maternal depressive symptoms, serum folate status, and pregnancy outcome: results of the Amsterdam BornChildren and their Development study.

[Van Dijk AE](http://www.ncbi.nlm.nih.gov/pubmed/?term=Van%20Dijk%20AE%5BAuthor%5D&cauthor=true&cauthor_uid=20833384)1, [Van Eijsden M](http://www.ncbi.nlm.nih.gov/pubmed/?term=Van%20Eijsden%20M%5BAuthor%5D&cauthor=true&cauthor_uid=20833384), [Stronks K](http://www.ncbi.nlm.nih.gov/pubmed/?term=Stronks%20K%5BAuthor%5D&cauthor=true&cauthor_uid=20833384), [Gemke RJ](http://www.ncbi.nlm.nih.gov/pubmed/?term=Gemke%20RJ%5BAuthor%5D&cauthor=true&cauthor_uid=20833384), [Vrijkotte TG](http://www.ncbi.nlm.nih.gov/pubmed/?term=Vrijkotte%20TG%5BAuthor%5D&cauthor=true&cauthor_uid=20833384).

### [Author information](http://www.ncbi.nlm.nih.gov/pubmed/?term=Maternal+depressive+symptoms%2C+serum+folate+status%2C+and+pregnancy+outcome%3A+results+of+the+Amsterdam+Born+Children+and+their+Development+study.)

### Abstract

#### OBJECTIVE:

The objective was to explore whether different levels of depressive symptoms in pregnant women and their serum folate statuscombined were associated with the gestational age and birthweight of their offspring.

#### STUDY DESIGN:

Data were derived from pregnant women in Amsterdam who completed a questionnaire covering depressive symptoms(Center for Epidemiological Studies Depression Scale) and from whom blood samples were taken to determine serum folate status. Only live-born singletons were included (n = 4044) in the multivariate regression analysis.

#### RESULTS:

When adjusted for potential confounders, only the association between major depressive symptoms and gestational age remained significant (-0.2 weeks; 95% confidence interval, -0.4 to -0.1). Women with depressive symptoms and low folate status (7.6%) experienced the shortest gestational age (38.6 weeks) and lowest birthweight (3270 g) (there was no significant interaction).

#### CONCLUSION:

Depressive symptoms were associated with shorter gestational age and related lower birthweight. The study results underline the importance of folic acid intake specifically in women suffering from depressive symptoms.

Exclusion reasons: No autism data.

28. [Childs Nerv Syst.](http://www.ncbi.nlm.nih.gov/pubmed/?term=Case+control+study+of+periconceptional+folic+acid+intake+and+nervous+system+tumors+in+children.) 2010 Dec;26(12):1727-33. doi: 10.1007/s00381-010-1187-x. Epub 2010 May 23.

# Case control study of periconceptional folic acid intake and nervous system tumors in children.

[Ortega-García JA](http://www.ncbi.nlm.nih.gov/pubmed/?term=Ortega-Garc%C3%ADa%20JA%5BAuthor%5D&cauthor=true&cauthor_uid=20496070)1, [Ferrís-Tortajada J](http://www.ncbi.nlm.nih.gov/pubmed/?term=Ferr%C3%ADs-Tortajada%20J%5BAuthor%5D&cauthor=true&cauthor_uid=20496070), [Claudio L](http://www.ncbi.nlm.nih.gov/pubmed/?term=Claudio%20L%5BAuthor%5D&cauthor=true&cauthor_uid=20496070), [Soldin OP](http://www.ncbi.nlm.nih.gov/pubmed/?term=Soldin%20OP%5BAuthor%5D&cauthor=true&cauthor_uid=20496070), [Sanchez-Sauco MF](http://www.ncbi.nlm.nih.gov/pubmed/?term=Sanchez-Sauco%20MF%5BAuthor%5D&cauthor=true&cauthor_uid=20496070), [Fuster-Soler JL](http://www.ncbi.nlm.nih.gov/pubmed/?term=Fuster-Soler%20JL%5BAuthor%5D&cauthor=true&cauthor_uid=20496070), [Martínez-Lage JF](http://www.ncbi.nlm.nih.gov/pubmed/?term=Mart%C3%ADnez-Lage%20JF%5BAuthor%5D&cauthor=true&cauthor_uid=20496070).

### [Author information](http://www.ncbi.nlm.nih.gov/pubmed/?term=Case+control+study+of+periconceptional+folic+acid+intake+and+nervous+system+tumors+in+children.)

### Abstract

#### PURPOSE:

Since 1992, the Centers for Disease Control and Prevention recommends that women of childbearing age consume 400 µg of folicacid per day to reduce the risk of neural tube defects (NTD). It has been speculated that both NTD and nervous system tumors (NST) may share common mechanisms of altered development. It examines the association between folic acid supplementation and the risk for childhood NST.

#### METHODS:

Incident cases of children with cancer in Spain registered between 2004 and 2006 were identified through the MACAPE Network Group. Tumors were classified as tumors derived from the neuroectoderm (cases) and those with a mesoderm origin (controls). In a second analysis, NST were further divided into central nervous system tumors (CNST) and sympathetic nervous system tumors (SNST). We compared folic acid supplementation between the groups.

#### RESULTS:

Overall, folic acid supplementation any time during pregnancy was similar between cases and controls (odds ratio (OR)=1.05; 95% confidence interval (CI) 0.92-1.20). However, supplementation before the 21st and 36th days of gestation resulted in significantly lower NST than inchildren with mesoderm tumors (OR=0.34; 95% CI 0.17-0.69 and OR=0.58; 95% CI 0.37-0.91, respectively). Preconceptional intakes of folicacid were also lower in NST although marginally nonsignificant (OR=0.44; 95% CI 0.10-1.02). When NST were divided into CNST and SNST, significant differences between tumors of mesoderm origin were only found for CNST.

#### CONCLUSIONS:

Our results support the hypothesis that folate supplementation reduces the risk of childhood NST, especially CNST. The specific mechanism and cellular role that folate may play in the development of CNST have yet to be elucidated.

Exclusion reasons: No autism data.

29. [Am J Med Genet B Neuropsychiatr Genet.](http://www.ncbi.nlm.nih.gov/pubmed/?term=A+functional+polymorphism+in+the+reduced+folate+carrier+gene+and+DNA+hypomethylation+in+mothers+of+children+with+autism.) 2010 Sep;153B(6):1209-20. doi: 10.1002/ajmg.b.31094.

A functional polymorphism in the reduced folate carrier gene and DNA hypomethylation in mothers of childrenwith autism.

[James SJ](http://www.ncbi.nlm.nih.gov/pubmed/?term=James%20SJ%5BAuthor%5D&cauthor=true&cauthor_uid=20468076)1, [Melnyk S](http://www.ncbi.nlm.nih.gov/pubmed/?term=Melnyk%20S%5BAuthor%5D&cauthor=true&cauthor_uid=20468076), [Jernigan S](http://www.ncbi.nlm.nih.gov/pubmed/?term=Jernigan%20S%5BAuthor%5D&cauthor=true&cauthor_uid=20468076), [Pavliv O](http://www.ncbi.nlm.nih.gov/pubmed/?term=Pavliv%20O%5BAuthor%5D&cauthor=true&cauthor_uid=20468076), [Trusty T](http://www.ncbi.nlm.nih.gov/pubmed/?term=Trusty%20T%5BAuthor%5D&cauthor=true&cauthor_uid=20468076), [Lehman S](http://www.ncbi.nlm.nih.gov/pubmed/?term=Lehman%20S%5BAuthor%5D&cauthor=true&cauthor_uid=20468076), [Seidel L](http://www.ncbi.nlm.nih.gov/pubmed/?term=Seidel%20L%5BAuthor%5D&cauthor=true&cauthor_uid=20468076), [Gaylor DW](http://www.ncbi.nlm.nih.gov/pubmed/?term=Gaylor%20DW%5BAuthor%5D&cauthor=true&cauthor_uid=20468076), [Cleves MA](http://www.ncbi.nlm.nih.gov/pubmed/?term=Cleves%20MA%5BAuthor%5D&cauthor=true&cauthor_uid=20468076).

[Author information](http://www.ncbi.nlm.nih.gov/pubmed/?term=A+functional+polymorphism+in+the+reduced+folate+carrier+gene+and+DNA+hypomethylation+in+mothers+of+children+with+autism.)

Abstract

The biologic basis of autism is complex and is thought to involve multiple and variable gene-environment interactions. While the logical focus has been on the affected child, the impact of maternal genetics on intrauterine microenvironment during pivotal developmental windows could be substantial. Folate-dependent one carbon metabolism is a highly polymorphic pathway that regulates the distribution of one-carbon derivatives between DNA synthesis (proliferation) and DNA methylation (cell-specific gene expression and differentiation). These pathways are essential to support the programmed shifts between proliferation and differentiation during embryogenesis and organogenesis. Maternal genetic variants that compromise intrauterine availability of folate derivatives could alter fetal cell trajectories and disrupt normal neurodevelopment. In this investigation, the frequency of common functional polymorphisms in the folate pathway was investigated in a large population-based sample ofautism case-parent triads. In case-control analysis, a significant increase in the reduced folate carrier (RFC1) G allele frequency was found among case mothers, but not among fathers or affected children. Subsequent log linear analysis of the RFC1 A80G genotype within family trios revealed that the maternal G allele was associated with a significant increase in risk of autism whereas the inherited genotype of the child was not. Further, maternal DNA from the autism mothers was found to be significantly hypomethylated relative to reference control DNA. Metabolic profiling indicated that plasma homocysteine, adenosine, and S-adenosylhomocyteine were significantly elevated among autism mothersconsistent with reduced methylation capacity and DNA hypomethylation. Together, these results suggest that the maternal genetics/epigenetics may influence fetal predisposition to autism.

Exclusion reasons: Review

30. [Br J Nutr.](http://www.ncbi.nlm.nih.gov/pubmed/?term=Undernutrition%2C+fatty+acid+and+micronutrient+status+in+relation+to+cognitive+performance+in+Indian+school+children%3A+a+cross-sectional+study) 2010 Apr;103(7):1056-64. doi: 10.1017/S000711450999273X. Epub 2009 Dec 14.

# Undernutrition, fatty acid and micronutrient status in relation to cognitive performance in Indian schoolchildren: a cross-sectional study.

[Eilander A](http://www.ncbi.nlm.nih.gov/pubmed/?term=Eilander%20A%5BAuthor%5D&cauthor=true&cauthor_uid=20003612)1, [Muthayya S](http://www.ncbi.nlm.nih.gov/pubmed/?term=Muthayya%20S%5BAuthor%5D&cauthor=true&cauthor_uid=20003612), [van der Knaap H](http://www.ncbi.nlm.nih.gov/pubmed/?term=van%20der%20Knaap%20H%5BAuthor%5D&cauthor=true&cauthor_uid=20003612), [Srinivasan K](http://www.ncbi.nlm.nih.gov/pubmed/?term=Srinivasan%20K%5BAuthor%5D&cauthor=true&cauthor_uid=20003612), [Thomas T](http://www.ncbi.nlm.nih.gov/pubmed/?term=Thomas%20T%5BAuthor%5D&cauthor=true&cauthor_uid=20003612), [Kok FJ](http://www.ncbi.nlm.nih.gov/pubmed/?term=Kok%20FJ%5BAuthor%5D&cauthor=true&cauthor_uid=20003612), [Kurpad AV](http://www.ncbi.nlm.nih.gov/pubmed/?term=Kurpad%20AV%5BAuthor%5D&cauthor=true&cauthor_uid=20003612), [Osendarp SJ](http://www.ncbi.nlm.nih.gov/pubmed/?term=Osendarp%20SJ%5BAuthor%5D&cauthor=true&cauthor_uid=20003612).

### [Author information](http://www.ncbi.nlm.nih.gov/pubmed/?term=Undernutrition%2C+fatty+acid+and+micronutrient+status+in+relation+to+cognitive+performance+in+Indian+school+children%3A+a+cross-sectional+study)

### Abstract

While undernutrition and anaemia have previously been linked to poor development of children, relatively little is known about the role of B-vitamins and fatty acids on cognition. The present study aims to explore the associations between indicators of body size, fatty acid andmicronutrient status on cognitive performance in 598 Indian school children aged 6-10 years. Baseline data of a clinical study were used to assess these associations by analyses of variance adjusting for age, sex, school, maternal education and cognitive tester. The Kaufman Assessment Battery for Children II was used to measure four cognitive domains, including fluid reasoning, short-term memory, retrieval ability and cognitive speediness. Scores were combined into an overall measure, named mental processing index (MPI). Body size indicators and Hb concentrations were significantly positively related to cognitive domains and MPI, such that increases of 1 sd in height-for-age and weight-for-age z-scores would each translate into a 0.09 sd increase in MPI, P = 0.0006 and 0.002, respectively. A 10 g/l increase in Hb concentrations would translate into a 0.08 sd increase in MPI, P = 0.0008. Log-transformed vitamin B12 concentrations were significantly inversely associated with short-term memory, retrieval ability and MPI (beta (95 % CI) = - 0.124 (- 0.224, - 0.023), P = 0.02). Other indicators of Fe, iodine, folate and fatty acidstatus were not significantly related to cognition. Our findings for body size, fatty acids and micronutrients were in agreement with previous observational studies. The inverse association of vitamin B12 with mental development was unexpected and needed further study.

Exclusion reasons: No folic acid data

31. [CMAJ.](http://www.ncbi.nlm.nih.gov/pubmed/?term=Effects+of+prenatal+multimicronutrient+supplementation+on+pregnancy+outcomes%3A+a+meta-analysis) 2009 Jun 9;180(12):E99-108. doi: 10.1503/cmaj.081777.

# Effects of prenatal multimicronutrient supplementation on pregnancy outcomes: a meta-analysis.

[Shah PS](http://www.ncbi.nlm.nih.gov/pubmed/?term=Shah%20PS%5BAuthor%5D&cauthor=true&cauthor_uid=19506270)1, [Ohlsson A](http://www.ncbi.nlm.nih.gov/pubmed/?term=Ohlsson%20A%5BAuthor%5D&cauthor=true&cauthor_uid=19506270); [Knowledge Synthesis Group on Determinants of Low Birth Weight and Preterm Births](http://www.ncbi.nlm.nih.gov/pubmed/?term=Knowledge%20Synthesis%20Group%20on%20Determinants%20of%20Low%20Birth%20Weight%20and%20Preterm%20Births%5BCorporate%20Author%5D).

### [Collaborators (11)](http://www.ncbi.nlm.nih.gov/pubmed/?term=Effects+of+prenatal+multimicronutrient+supplementation+on+pregnancy+outcomes%3A+a+meta-analysis)

### [Author information](http://www.ncbi.nlm.nih.gov/pubmed/?term=Effects+of+prenatal+multimicronutrient+supplementation+on+pregnancy+outcomes%3A+a+meta-analysis)

### Abstract

#### BACKGROUND:

Reduced intake of micronutrients during pregnancy exposes women to nutritional deficiencies and may affect fetal growth. We conducted a systematic review to examine the efficacy of prenatal supplementation with multimicronutrients on pregnancy outcomes.

#### METHODS:

We searched MEDLINE, EMBASE, CINAHL and the Cochrane Library for relevant articles published in English up to December 2008. We also searched the bibliographies of selected articles as well as clinical trial registries. The primary outcome was low birth weight; secondaryoutcomes were preterm birth, small-for-gestational-age infants, birth weight and gestational age.

#### RESULTS:

We observed a significant reduction in the risk of low birth weight among infants born to women who received multimicronutrients during pregnancy compared with placebo (relative risk [RR] 0.81, 95% confidence interval [CI] 0.73-0.91) or iron-folic acid supplementation (RR 0.83, 95% CI 0.74-0.93). Birth weight was significantly higher among infants whose mothers were in the multimicronutrient group than among those whose mothers received iron-folic acid supplementation (weighted mean difference 54 g, 95% CI 36 g-72 g). There was no significant differences in the risk of preterm birth or small-for-gestational-age infants between the 3 study groups.

#### INTERPRETATION:

Prenatal multimicronutrient supplementation was associated with a significantly reduced risk of low birth weight and with improved birth weight when compared with iron-folic acid supplementation. There was no significant effect of multimicronutrientsupplementation on the risk of preterm birth or small-for-gestational-age infants.

Exclusion reasons: Review

32. [N Engl J Med.](http://www.ncbi.nlm.nih.gov/pubmed/19369666) 2009 Apr 16;360(16):1597-605. doi: 10.1056/NEJMoa0803531.

# Cognitive function at 3 years of age after fetal exposure to antiepileptic drugs.

[Meador KJ](http://www.ncbi.nlm.nih.gov/pubmed/?term=Meador%20KJ%5BAuthor%5D&cauthor=true&cauthor_uid=19369666), [Baker GA](http://www.ncbi.nlm.nih.gov/pubmed/?term=Baker%20GA%5BAuthor%5D&cauthor=true&cauthor_uid=19369666), [Browning N](http://www.ncbi.nlm.nih.gov/pubmed/?term=Browning%20N%5BAuthor%5D&cauthor=true&cauthor_uid=19369666), [Clayton-Smith J](http://www.ncbi.nlm.nih.gov/pubmed/?term=Clayton-Smith%20J%5BAuthor%5D&cauthor=true&cauthor_uid=19369666), [Combs-Cantrell DT](http://www.ncbi.nlm.nih.gov/pubmed/?term=Combs-Cantrell%20DT%5BAuthor%5D&cauthor=true&cauthor_uid=19369666), [Cohen M](http://www.ncbi.nlm.nih.gov/pubmed/?term=Cohen%20M%5BAuthor%5D&cauthor=true&cauthor_uid=19369666), [Kalayjian LA](http://www.ncbi.nlm.nih.gov/pubmed/?term=Kalayjian%20LA%5BAuthor%5D&cauthor=true&cauthor_uid=19369666), [Kanner A](http://www.ncbi.nlm.nih.gov/pubmed/?term=Kanner%20A%5BAuthor%5D&cauthor=true&cauthor_uid=19369666), [Liporace JD](http://www.ncbi.nlm.nih.gov/pubmed/?term=Liporace%20JD%5BAuthor%5D&cauthor=true&cauthor_uid=19369666), [Pennell PB](http://www.ncbi.nlm.nih.gov/pubmed/?term=Pennell%20PB%5BAuthor%5D&cauthor=true&cauthor_uid=19369666), [Privitera M](http://www.ncbi.nlm.nih.gov/pubmed/?term=Privitera%20M%5BAuthor%5D&cauthor=true&cauthor_uid=19369666), [Loring DW](http://www.ncbi.nlm.nih.gov/pubmed/?term=Loring%20DW%5BAuthor%5D&cauthor=true&cauthor_uid=19369666);[NEAD Study Group](http://www.ncbi.nlm.nih.gov/pubmed/?term=NEAD%20Study%20Group%5BCorporate%20Author%5D).

### [Collaborators (102)](http://www.ncbi.nlm.nih.gov/pubmed/19369666)

### Abstract

#### BACKGROUND:

Fetal exposure of animals to antiepileptic drugs at doses lower than those required to produce congenital malformations can produce cognitive and behavioral abnormalities, but cognitive effects of fetal exposure of humans to antiepileptic drugs are uncertain.

#### METHODS:

Between 1999 and 2004, we enrolled pregnant women with epilepsy who were taking a single antiepileptic agent (carbamazepine, lamotrigine, phenytoin, or valproate) in a prospective, observational, multicenter study in the United States and the United Kingdom. The primary analysis is a comparison of neurodevelopmental outcomes at the age of 6 years after exposure to different antiepileptic drugs in utero. This report focuses on a planned interim analysis of cognitive outcomes in 309 children at 3 years of age.

#### RESULTS:

At 3 years of age, children who had been exposed to valproate in utero had significantly lower IQ scores than those who had been exposed to other antiepileptic drugs. After adjustment for maternal IQ, maternal age, antiepileptic-drug dose, gestational age at birth, and maternal preconception use of folate, the mean IQ was 101 for children exposed to lamotrigine, 99 for those exposed to phenytoin, 98 for those exposed to carbamazepine, and 92 for those exposed to valproate. On average, children exposed to valproate had an IQ score 9 points lower than the score of those exposed to lamotrigine (95% confidence interval [CI], 3.1 to 14.6; P=0.009), 7 points lower than the score of those exposed to phenytoin (95% CI, 0.2 to 14.0; P=0.04), and 6 points lower than the score of those exposed to carbamazepine (95% CI, 0.6 to 12.0; P=0.04). The association between valproate use and IQ was dose dependent. Children's IQs were significantly related to maternal IQs among children exposed to carbamazepine, lamotrigine, or phenytoin but not among those exposed to valproate.

#### CONCLUSIONS:

In utero exposure to valproate, as compared with other commonly used antiepileptic drugs, is associated with an increased risk of impaired cognitive function at 3 years of age. This finding supports a recommendation that valproate not be used as a first-choice drug in women of childbearing potential.

Exclusion reason: No folic acid data.

33. [Pediatrics.](http://www.ncbi.nlm.nih.gov/pubmed/?term=Effects+of+maternal+multimicronutrient+supplementation+on+the+mental+development++of+infants+in+rural+western+China%3A+follow-up+evaluation+of+a+double-blind%2C+randomized%2C+controlled+trial.) 2009 Apr;123(4):e685-92. doi: 10.1542/peds.2008-3007.

# Effects of maternal multimicronutrient supplementation on the mental development of infants in rural westernChina: follow-up evaluation of a double-blind, randomized, controlled trial.

[Li Q](http://www.ncbi.nlm.nih.gov/pubmed/?term=Li%20Q%5BAuthor%5D&cauthor=true&cauthor_uid=19336358)1, [Yan H](http://www.ncbi.nlm.nih.gov/pubmed/?term=Yan%20H%5BAuthor%5D&cauthor=true&cauthor_uid=19336358), [Zeng L](http://www.ncbi.nlm.nih.gov/pubmed/?term=Zeng%20L%5BAuthor%5D&cauthor=true&cauthor_uid=19336358), [Cheng Y](http://www.ncbi.nlm.nih.gov/pubmed/?term=Cheng%20Y%5BAuthor%5D&cauthor=true&cauthor_uid=19336358), [Liang W](http://www.ncbi.nlm.nih.gov/pubmed/?term=Liang%20W%5BAuthor%5D&cauthor=true&cauthor_uid=19336358), [Dang S](http://www.ncbi.nlm.nih.gov/pubmed/?term=Dang%20S%5BAuthor%5D&cauthor=true&cauthor_uid=19336358), [Wang Q](http://www.ncbi.nlm.nih.gov/pubmed/?term=Wang%20Q%5BAuthor%5D&cauthor=true&cauthor_uid=19336358), [Tsuji I](http://www.ncbi.nlm.nih.gov/pubmed/?term=Tsuji%20I%5BAuthor%5D&cauthor=true&cauthor_uid=19336358).

### [Author information](http://www.ncbi.nlm.nih.gov/pubmed/?term=Effects+of+maternal+multimicronutrient+supplementation+on+the+mental+development++of+infants+in+rural+western+China%3A+follow-up+evaluation+of+a+double-blind%2C+randomized%2C+controlled+trial.)

### Abstract

#### OBJECTIVE:

We investigated the benefits of maternal multimicronutrient supplementation during gestation on the mental and psychomotordevelopment of infants.

#### METHODS:

In a double-blind, randomized, controlled trial, pregnant women (N = 5828) in 2 rural counties in western China were assigned randomly to receive multimicronutrient (5 minerals and 10 vitamins at levels approximating the recommended daily allowance), folic acid plus iron, or folic acid supplementation daily from approximately 14 weeks of gestation until delivery. We assessed a subset of the newborns (N = 1305) from the 3 supplementation groups by measuring their mental and psychomotor development with the Bayley Scales of InfantDevelopment, at 3, 6, and 12 months of age. Multilevel analyses were used to compare the mental development and psychomotor developmentraw scores at 3, 6, and 12 months.

#### RESULTS:

Multimicronutrient supplementation was associated with mean increases in mental development raw scores for infants at 1 year of age of 1.00 and 1.22 points, compared with folic acid only and folic acid plus iron supplementation, respectively. However, supplementationdid not increase significantly the psychomotor development raw scores up to 1 year of age.

#### CONCLUSION:

Compared with iron and folic acid supplementation, the administration of multimicronutrients to pregnant women improved themental development of their children at 1 year of age.

Exclusion reasons: No folic acid data

34. [Food Nutr Bull.](http://www.ncbi.nlm.nih.gov/pubmed/18709885) 2008 Jun;29(2 Suppl):S101-11; discussion S112-5.

# Effects of folate and vitamin B12 deficiencies during pregnancy on fetal, infant, and child development.

[Molloy AM](http://www.ncbi.nlm.nih.gov/pubmed/?term=Molloy%20AM%5BAuthor%5D&cauthor=true&cauthor_uid=18709885)1, [Kirke PN](http://www.ncbi.nlm.nih.gov/pubmed/?term=Kirke%20PN%5BAuthor%5D&cauthor=true&cauthor_uid=18709885), [Brody LC](http://www.ncbi.nlm.nih.gov/pubmed/?term=Brody%20LC%5BAuthor%5D&cauthor=true&cauthor_uid=18709885), [Scott JM](http://www.ncbi.nlm.nih.gov/pubmed/?term=Scott%20JM%5BAuthor%5D&cauthor=true&cauthor_uid=18709885), [Mills JL](http://www.ncbi.nlm.nih.gov/pubmed/?term=Mills%20JL%5BAuthor%5D&cauthor=true&cauthor_uid=18709885).

### [Author information](http://www.ncbi.nlm.nih.gov/pubmed/18709885)

### Abstract

The importance of folate in reproduction can be appreciated by considering that the existence of the vitamin was first suspected from efforts to explain a potentially fatal megaloblastic anemia in young pregnant women in India. Today, low maternal folate status during pregnancy and lactation remains a significant cause of maternal morbidity in some communities. The folate status of the neonate tends to be protected at the expense of maternal stores; nevertheless, there is mounting evidence that inadequate maternal folate status during pregnancy may lead to lowinfant birthweight, thereby conferring risk of developmental and long-term adverse health outcomes. Moreover, folate-related anemia during childhood and adolescence might predispose children to further infections and disease. The role of folic acid in prevention of neural tube defects (NTD) is now established, and several studies suggest that this protection may extend to some other birth defects. In terms of maternal health, clinical vitamin B12 deficiency may be a cause of infertility or recurrent spontaneous abortion. Starting pregnancy with an inadequate vitamin B12status may increase risk of birth defects such as NTD, and may contribute to preterm delivery, although this needs further evaluation. Furthermore, inadequate vitamin B12 status in the mother may lead to frank deficiency in the infant if sufficient fetal stores of vitamin B12 are not laid down during pregnancy or are not available in breastmilk. However, the implications of starting pregnancy and lactation with low vitamin B12 status have not been sufficiently researched.

Exclusion reasons: review

35. [Epilepsy Behav.](http://www.ncbi.nlm.nih.gov/pubmed/18346940) 2008 Jul;13(1):229-36. doi: 10.1016/j.yebeh.2008.01.010. Epub 2008 Mar 17.

# Motor and mental development of infants exposed to antiepileptic drugs in utero.

[Thomas SV](http://www.ncbi.nlm.nih.gov/pubmed/?term=Thomas%20SV%5BAuthor%5D&cauthor=true&cauthor_uid=18346940)1, [Ajaykumar B](http://www.ncbi.nlm.nih.gov/pubmed/?term=Ajaykumar%20B%5BAuthor%5D&cauthor=true&cauthor_uid=18346940), [Sindhu K](http://www.ncbi.nlm.nih.gov/pubmed/?term=Sindhu%20K%5BAuthor%5D&cauthor=true&cauthor_uid=18346940), [Nair MK](http://www.ncbi.nlm.nih.gov/pubmed/?term=Nair%20MK%5BAuthor%5D&cauthor=true&cauthor_uid=18346940), [George B](http://www.ncbi.nlm.nih.gov/pubmed/?term=George%20B%5BAuthor%5D&cauthor=true&cauthor_uid=18346940), [Sarma PS](http://www.ncbi.nlm.nih.gov/pubmed/?term=Sarma%20PS%5BAuthor%5D&cauthor=true&cauthor_uid=18346940).

### [Author information](http://www.ncbi.nlm.nih.gov/pubmed/18346940)

### Abstract

We prospectively evaluated the mental (MeDQ) and motor (MoDQ) developmental quotients of 395 (67.5% of the eligible) infants of mothers with epilepsy (IME) (mean age: 15 months) enrolled in the Kerala Registry of Epilepsy and Pregnancy between 1998 and 2004. The same developmental pediatricians, blinded to antiepileptic drug (AED) exposure, evaluated the children using the Indian adaptation of the Bayley Scale of Infant Development: Their mean MeDQ was 89.1+/-29.9 and mean MoDQ was 90.7+/-26.9. The MeDQ and MoDQ were impaired (<84) for 150 (37.6%) and 133 (33.5%) IME, respectively. Maternal age, type of epilepsy, seizure frequency, or use of folic acid did not correlate with the mean MeDQ or MoDQ. Maternal education was significantly correlated with the MoDQ, but not with the MeDQ, of the infants. Infants not exposed to AEDs (n=32) had a higher MeDQ (mean: 92.3, 95% CI: 81.4-103.2) and MoDQ (mean 94.7; 95% CI 84.9-104.5) than those exposed to AEDs (MeDQ--mean: 88.6, 95% CI: 85.5-91.6; MoDQ--mean: 90.0, 95% CI: 87.3-92.8). Those exposed to polytherapy had significantly lower developmental quotients than those exposed to monotherapy. Cumulative AED scores during pregnancy had an inverse relationship with developmental quotients. On multiple regression analysis, polytherapy was a stronger predictor of lower developmental quotients than dosage. Compared with carbamazepine monotherapy, valproate monotherapy was associated with significantly lower MeDQ and MoDQ in IME (93.1 and 95 vs 86.9 and 86.1), but the differences between other AEDs were not significant for IME exposed to valproate monotherapy. A limitation of the study is that the influence of maternal intelligence on developmental quotients was not evaluated.

Exclusion reasons: No folic acid data.

36. [J Autism Dev Disord.](http://www.ncbi.nlm.nih.gov/pubmed/?term=Brief+report%3A+autistic+symptoms%2C+developmental+regression%2C+mental+retardation%2C+epilepsy%2C+and+dyskinesias+in+CNS+folate+deficiency.) 2008 Jul;38(6):1170-7. Epub 2007 Nov 20.

# Brief report: autistic symptoms, developmental regression, mental retardation, epilepsy, and dyskinesias in CNS folate deficiency.

[Moretti P](http://www.ncbi.nlm.nih.gov/pubmed/?term=Moretti%20P%5BAuthor%5D&cauthor=true&cauthor_uid=18027081)1, [Peters SU](http://www.ncbi.nlm.nih.gov/pubmed/?term=Peters%20SU%5BAuthor%5D&cauthor=true&cauthor_uid=18027081), [Del Gaudio D](http://www.ncbi.nlm.nih.gov/pubmed/?term=Del%20Gaudio%20D%5BAuthor%5D&cauthor=true&cauthor_uid=18027081), [Sahoo T](http://www.ncbi.nlm.nih.gov/pubmed/?term=Sahoo%20T%5BAuthor%5D&cauthor=true&cauthor_uid=18027081), [Hyland K](http://www.ncbi.nlm.nih.gov/pubmed/?term=Hyland%20K%5BAuthor%5D&cauthor=true&cauthor_uid=18027081), [Bottiglieri T](http://www.ncbi.nlm.nih.gov/pubmed/?term=Bottiglieri%20T%5BAuthor%5D&cauthor=true&cauthor_uid=18027081), [Hopkin RJ](http://www.ncbi.nlm.nih.gov/pubmed/?term=Hopkin%20RJ%5BAuthor%5D&cauthor=true&cauthor_uid=18027081), [Peach E](http://www.ncbi.nlm.nih.gov/pubmed/?term=Peach%20E%5BAuthor%5D&cauthor=true&cauthor_uid=18027081), [Min SH](http://www.ncbi.nlm.nih.gov/pubmed/?term=Min%20SH%5BAuthor%5D&cauthor=true&cauthor_uid=18027081), [Goldman D](http://www.ncbi.nlm.nih.gov/pubmed/?term=Goldman%20D%5BAuthor%5D&cauthor=true&cauthor_uid=18027081), [Roa B](http://www.ncbi.nlm.nih.gov/pubmed/?term=Roa%20B%5BAuthor%5D&cauthor=true&cauthor_uid=18027081), [Bacino CA](http://www.ncbi.nlm.nih.gov/pubmed/?term=Bacino%20CA%5BAuthor%5D&cauthor=true&cauthor_uid=18027081), [Scaglia F](http://www.ncbi.nlm.nih.gov/pubmed/?term=Scaglia%20F%5BAuthor%5D&cauthor=true&cauthor_uid=18027081).

### [Author information](http://www.ncbi.nlm.nih.gov/pubmed/?term=Brief+report%3A+autistic+symptoms%2C+developmental+regression%2C+mental+retardation%2C+epilepsy%2C+and+dyskinesias+in+CNS+folate+deficiency.)

### Abstract

We studied seven children with CNS folate deficiency (CFD). All cases exhibited psychomotor retardation, regression, cognitive delay, and dyskinesia; six had seizures; four demonstrated neurological abnormalities in the neonatal period. Two subjects had profound neurological abnormalities that precluded formal behavioral testing. Five subjects received ADOS and ADI-R testing and met diagnostic criteria for autism or autism spectrum disorders. They exhibited difficulties with transitions, insistence on sameness, unusual sensory interests, and repetitive behaviors. Those with the best language skills largely used repetitive phrases. No mutations were found in folate transporter or folate enzyme genes. These findings demonstrate that autistic features are salient in CFD and suggest that a subset of children with developmental regression, mentalretardation, seizures, dyskinesia, and autism may have CNS folate abnormalities.

Exclusion reasons: No folic acid data

37.[Hum Reprod.](http://www.ncbi.nlm.nih.gov/pubmed/?term=Do+children+born+after+assisted+conception+have+less+risk+of+developing+infantile+autism%3F) 2007 Jul;22(7):1841-3. Epub 2007 Apr 24.

# Do children born after assisted conception have less risk of developing infantile autism?

[Maimburg RD](http://www.ncbi.nlm.nih.gov/pubmed/?term=Maimburg%20RD%5BAuthor%5D&cauthor=true&cauthor_uid=17456530)1, [Vaeth M](http://www.ncbi.nlm.nih.gov/pubmed/?term=Vaeth%20M%5BAuthor%5D&cauthor=true&cauthor_uid=17456530).

### [Author information](http://www.ncbi.nlm.nih.gov/pubmed/?term=Do+children+born+after+assisted+conception+have+less+risk+of+developing+infantile+autism%3F)

### Abstract

#### BACKGROUND:

A Danish population based matched case-control study of perinatal risk factors in children with infantile autism has provided some interesting and surprising observations regarding infantile autism and children born after assisted conception.

#### METHODS AND RESULTS:

The cases (461) consisted of all children born between 1990 and 1999 and diagnosed with infantile autism in the Danish Psychiatric Central Register before February 2001. Matched controls were identified in the Danish Civil Registration System. The main exposure measures included obstetric risk factors for infantile autism. We found a 59% decreased risk for developing infantile autism among children conceived after assisted conception (odds ratio [OR] 0.41, 95% [0.19-0.89]) and a 63% decreased risk after adjusting for known risk factors for assisted conception and infantile autism (OR 0.37, 95% [0.14-0.98]).

#### CONCLUSION:

We found that children born after assisted conception had a lower risk of developing infantile autism then their matched controls. Our observations could possibly be explained by the mother's health status before and during early pregnancy. Our findings require further investigation in larger studies.

Exclusion reasons: No folic acid data

38. [Harv Rev Psychiatry.](http://www.ncbi.nlm.nih.gov/pubmed/?term=Do+maternal+folate+and+homocysteine+levels+play+a+role+in+neurodevelopmental+processes+that+increase+risk+for+schizophrenia%3F) 2005 Jul-Aug;13(4):197-205.

# Do maternal folate and homocysteine levels play a role in neurodevelopmental processes that increase risk forschizophrenia?

[Picker JD](http://www.ncbi.nlm.nih.gov/pubmed/?term=Picker%20JD%5BAuthor%5D&cauthor=true&cauthor_uid=16126606)1, [Coyle JT](http://www.ncbi.nlm.nih.gov/pubmed/?term=Coyle%20JT%5BAuthor%5D&cauthor=true&cauthor_uid=16126606).

### [Author information](http://www.ncbi.nlm.nih.gov/pubmed/?term=Do+maternal+folate+and+homocysteine+levels+play+a+role+in+neurodevelopmental+processes+that+increase+risk+for+schizophrenia%3F)

### Abstract

#### OBJECTIVE:

Evidence from many different lines of research supports the hypothesis that schizophrenia is a disorder of development with etiological factors implicated as early as the second trimester in utero. We suggest that low maternal folate, acting to increase homocysteinelevels, may provide a functional link between many of the identified prenatal risk factors and the hypothesized mechanisms wherebyneurodevelopmental patterning deviates toward a schizophrenic potential.

#### METHODS:

PubMed was searched from the present back to 1963, when elevated homocysteine was identified as a pathogen in homocystinuria as first described by Carson and colleagues (Arch Dis Child 1963;38:425-36). All articles for homocystinuria, homocysteine, folate, and development with schizophrenia were evaluated.

#### RESULTS:

The findings from this review support the hypothesis that maternal low folate and high homocysteine levels may provide a potential teratogenic mechanism that increases the risk for developing schizophrenia.

#### CONCLUSION:

The potential role of maternal folate deficiency and hyperhomocystinemia in the genesis of schizophrenia would extend the range of their known teratogenic effects. Given the potential for preventive treatment offered by this hypothesis, we believe further investigation into this mechanism is warranted.

Exclusion reasons: Review

39. [Am J Clin Nutr.](http://www.ncbi.nlm.nih.gov/pubmed/?term=Vitamin+supplementation+of+HIV-infected+women+improves+postnatal+child+growth.) 2005 Apr;81(4):880-8.

# Vitamin supplementation of HIV-infected women improves postnatal child growth.

[Villamor E](http://www.ncbi.nlm.nih.gov/pubmed/?term=Villamor%20E%5BAuthor%5D&cauthor=true&cauthor_uid=15817867)1, [Saathoff E](http://www.ncbi.nlm.nih.gov/pubmed/?term=Saathoff%20E%5BAuthor%5D&cauthor=true&cauthor_uid=15817867), [Bosch RJ](http://www.ncbi.nlm.nih.gov/pubmed/?term=Bosch%20RJ%5BAuthor%5D&cauthor=true&cauthor_uid=15817867), [Hertzmark E](http://www.ncbi.nlm.nih.gov/pubmed/?term=Hertzmark%20E%5BAuthor%5D&cauthor=true&cauthor_uid=15817867), [Baylin A](http://www.ncbi.nlm.nih.gov/pubmed/?term=Baylin%20A%5BAuthor%5D&cauthor=true&cauthor_uid=15817867), [Manji K](http://www.ncbi.nlm.nih.gov/pubmed/?term=Manji%20K%5BAuthor%5D&cauthor=true&cauthor_uid=15817867), [Msamanga G](http://www.ncbi.nlm.nih.gov/pubmed/?term=Msamanga%20G%5BAuthor%5D&cauthor=true&cauthor_uid=15817867), [Hunter DJ](http://www.ncbi.nlm.nih.gov/pubmed/?term=Hunter%20DJ%5BAuthor%5D&cauthor=true&cauthor_uid=15817867), [Fawzi WW](http://www.ncbi.nlm.nih.gov/pubmed/?term=Fawzi%20WW%5BAuthor%5D&cauthor=true&cauthor_uid=15817867).

### [Author information](http://www.ncbi.nlm.nih.gov/pubmed/?term=Vitamin+supplementation+of+HIV-infected+women+improves+postnatal+child+growth.)

### Abstract

#### BACKGROUND:

Linear growth retardation and wasting are common in children born to HIV-infected women. Inexpensive interventions that could improve the postnatal growth pattern of such children are needed.

#### OBJECTIVE:

The objective was to examine the effect of supplementing HIV-infected women with multivitamins or vitamin A and beta-carotene, during and after pregnancy, on the growth of their children during the first 2 y of life.

#### DESIGN:

We conducted a randomized placebo-controlled trial in 886 mother-infant pairs in Tanzania. At the first prenatal visit, HIV-infectedwomen were randomly assigned to 1 of 4 daily oral regimens in a 2 x 2 factorial fashion: multivitamins (MV: thiamine, riboflavin, vitamin B-6, niacin, vitamin B-12, vitamin C, vitamin E, and folic acid), preformed vitamin A + beta-carotene (VA/BC), MV including VA/BC, or placebo.Supplementation continued during the first 2 y postpartum and thereafter. Children were weighed and measured monthly, and all receivedvitamin A supplements after 6 mo of age per the standard of care.

#### RESULTS:

Multivitamins had a significant positive effect on attained weight (459 g; 95% CI: 35, 882; P = 0.03) and on weight-for-age (0.42; 95% CI: 0.07, 0.77; P = 0.02) and weight-for-length (0.38; 95% CI: 0.07, 0.68; P = 0.01) z scores at 24 mo. VA/BC seemed to reduce the benefits of MV on these outcomes. No significant effects were observed on length, midupper arm circumference, or head circumference.

#### CONCLUSION:

Supplementation of HIV-infected women with multivitamins (vitamin B complex, vitamin C, and vitamin E) during pregnancy and lactation is an effective intervention for improving ponderal growth in children.

Exclusion reason: No autism data

40. [Cochrane Database Syst Rev.](http://www.ncbi.nlm.nih.gov/pubmed/14584018) 2003;(4):CD004514.

# Folic acid with or without vitamin B12 for cognition and dementia.

[Malouf M](http://www.ncbi.nlm.nih.gov/pubmed/?term=Malouf%20M%5BAuthor%5D&cauthor=true&cauthor_uid=14584018)1, [Grimley EJ](http://www.ncbi.nlm.nih.gov/pubmed/?term=Grimley%20EJ%5BAuthor%5D&cauthor=true&cauthor_uid=14584018), [Areosa SA](http://www.ncbi.nlm.nih.gov/pubmed/?term=Areosa%20SA%5BAuthor%5D&cauthor=true&cauthor_uid=14584018).

### [Author information](http://www.ncbi.nlm.nih.gov/pubmed/14584018)

### Update in

- [Folic acid with or without vitamin B12 for the prevention and treatment of healthy elderly and demented people.](http://www.ncbi.nlm.nih.gov/pubmed/18843658) [Cochrane Database Syst Rev. 2008]

### Abstract

#### BACKGROUND:

Folates are vitamins essential to the development of the central nervous system. Insufficient folate activity at the time of conception and early pregnancy can result in congenital neural tube defects. In adult life folate deficiency has been known for decades to produce a characteristic form of anaemia ("megaloblastic"). More recently degrees of folate inadequacy, not severe enough to produce anaemia, have been found to be associated with high blood levels of the amino acid homocysteine. Such degrees of folate inadequacy can arise because of insufficient folates in the diet or because of inefficient absorption or metabolic utilisation of folates due to genetic variations. Conventional criteria for diagnosing folate deficiency may be inadequate for identifying people capable of benefiting from dietary supplementation. High blood levels of homocysteine have been linked with the risk of arterial disease, dementia and Alzheimer's disease. There is therefore interest in whether dietary supplements of folic acid (an artificial chemical analogue of naturally occurring folates) can improve cognitive function of people at risk of cognitive decline associated with ageing or dementia, whether by affecting homocysteine metabolism or through other mechanisms. There is a risk that iffolic acid is given to people who have undiagnosed deficiency of vitamin B12 it may lead to neurological damage. Vitamin B12 deficiency produces both an anaemia identical to that of folate deficiency but also causes irreversible damage to the central and peripheral nervous systems.Folic acid will correct the anaemia of vitamin B12 deficiency and so delay diagnosis but will not prevent progression to neurological damage. For this reason trials of folic acid supplements may involve simultaneous administration of vitamin B12. Apparent benefit from folic acid given in the combination would therefore need to be "corrected" for any effect of vitamin B12 alone. A separate Cochrane review of vitamin B12 and cognitive function is being prepared.

#### OBJECTIVES:

To examine the effects of folic acid supplementation, with or without vitamin B12, on elderly healthy and demented people, in preventing cognitive impairment or retarding its progress.

#### SEARCH STRATEGY:

Trials were identified from a search of the Cochrane Dementia and Cognitive Improvement Specialized Register Group on 9 April 2003 using the terms: folic acid, folate, vitamin B9, leucovorin, methyltetrahydrofolate, vitamin B12, cobalamin, cyanocobalamin,dementia, cognitive function, cognitive impairment, Alzheimer's disease, vascular dementia, mixed dementia and controlled trials. MEDLINE and EMBASE (both all years) were searched for additional trials on healthy people.

#### SELECTION CRITERIA:

All double-blind placebo-controlled randomized trials, in which supplements of folic acid with or without vitamin B12were compared with placebo for elderly healthy people or people with any type of dementia or cognitive impairment.

#### DATA COLLECTION AND ANALYSIS:

The reviewers independently applied the selection criteria and assessed study quality. One reviewer extracted and analysed the data. In comparing intervention with placebo, weighted mean differences, and standardized mean difference or odds ratios were estimated.

#### MAIN RESULTS:

Four randomized controlled trials fulfilled the inclusion criteria for this review. One trial (Bryan 2002) enrolled healthy women, and three (Fioravanti 1997; Sommer 1998; VITAL 2003) recruited people with mild to moderate cognitive impairment or dementia with or without diagnosed folate deficiency. Fioravanti 1997 enrolled people with mild to moderate cognitive impairment or dementia as judged by scores on the Mini-Mental State Examination (MMSE) and Global Deterioration Scale and with serum folate level<3ng/l. One trial (VITAL 2003) studied the effects of a combination of vitamin B12 and folic acid on patients with mild to moderate cognitive impairment due to Alzheimer's disease or mixeddementia. The analysis from the included trials found no benefit from folic acid with or without vitamin B12 in comparison with placebo on any measures of cognition and mood for healthy or cognitively impaired or demented people: Folic acid effect and healthy participants: there was no benefit from of oral 750 mcg folic acid per day for five weeks compared with placebo on measures of cognition and mood of 19 healthy women aged 65 to 92. Folic acid effect and people with mild to moderate cognitive decline or dementia: there were no statistically significant results in favour of folic acid with or without vitamin B12 on any measures of cognitive function. Scores on the Mini-Mental State Examination (MMSE) revealed no statistically significant benefit from 2 mg per day folic acid plus 1mg vitamin B12 for 12 weeks when compared with placebo (WMD 0.39, 95% CI -0.43 to 1.21, P=0.35). Cognitive scores on the Alzheimer's Disease Scale (ADAS-Cog) showed no statistically significant benefit from 2 mg /day folic acid plus 1 mg /day vitamin B12 for 12 weeks compared with placebo (WMD 0.41, 95% -1.25 to 2.07, P=4.63). The Bristol Activities of Daily Living Scale (BADL) revealed no benefit from 2mg per day of folic acid plus 1 mg vitamin B12 for 12 weeks in comparison with placebo (WMD -0.57, 95%CI -1.95 to 0.81, P=0.42). None of the sub tests of the Randt Memory Test (RMT) showed statistically significant benefit from 15 mg of folic acid orally per day for 9 weeks when compared with placebo. One trial (Sommer 1998) reported a significant decline compared with placebo in two cognitive function tasks in demented patients who had received high doses of folic acid (10 mg /day) for unspecified periods. One trial (VITAL 2003) showed that 2 mg folic acid plus 1 mg vitamin B12 daily for 12 weeks significantly lowered serum homocysteine concentrations (P <0.0001).

#### REVIEWER'S CONCLUSIONS:

There was no beneficial effect of 750 mcg of folic acid per day on measures of cognition or mood in older healthy women. In patients with mild to moderate cognitive decline and different forms of dementia there was no benefit from folic acid on measures of cognition or mood. Folic acid plus vitamin B12 was effective in reducing the serum homocysteine concentrations. Folic acid was well tolerated and no adverse effects were reported. More studies are needed.

Exclusion reasons: Review

41. [J Gend Specif Med.](http://www.ncbi.nlm.nih.gov/pubmed/?term=The+importance+of+folic+acid.+AND+Berg+M) 1999 May-Jun;2(3):24-8.

# The importance of folic acid.

[Berg MJ](http://www.ncbi.nlm.nih.gov/pubmed/?term=Berg%20MJ%5BAuthor%5D&cauthor=true&cauthor_uid=11252849)1.

### [Author information](http://www.ncbi.nlm.nih.gov/pubmed/?term=The+importance+of+folic+acid.+AND+Berg+M)

### Abstract

Folic acid is necessary for cell development; for the metabolism of specific biochemical reactions in the body, such as the conversion of homocysteine to methionine; and for the metabolism of specific anticonvulsant drugs. Folic acid has an interrelationship with vitamin B12. A deficiency of folate increases the risk of NTDs, as well as contributing to hyperhomocystinemia, a condition associated with increased cardiovascular disease and NTDs. For the prevention of NTDs, it is recommended that a woman of childbearing age consume a daily folate intake of 400 micrograms; however, the average dietary folate intake is half that amount, and the FDA folate fortification of cereal grains adds only 100 micrograms daily. The woman in her childbearing years does not meet the recommendation with dietary and food fortification. Periconceptionalfolic acid supplementation is essential, because the neural tube closes 23 to 27 days after conception. Therefore, a multiple vitamin containingfolic acid is the practical solution at present if the food fortification is not increased. The bioavailability of folate in the vitamin preparation is approximately double that of dietary folate. Most preparations contain 400 micrograms of folic acid, and if the woman took a multiple vitamin (400 micrograms of folate) in addition to her diet (230 micrograms of folate), she would not exceed 1000 micrograms (1 mg) daily, which is considered the upper limit of daily folate ingestion by dietary fortification and supplementation before the masking of vitamin B12 becomes a concern. However, in this group of patients, pernicious anemia is rare. Regarding cardiovascular disease in men and women, there are no long-term studies showing the benefit of folic acid in reducing the homocysteine level. At present, there are only estimations. However, they should not be ignored. Although it is not the current standard of practice, adding a multiple vitamin containing folic acid to the regimen of men and women starting anticonvulsant medication should be considered in order to prevent the folate lowering observed with such commonly used drugs as PHT and carbamazepine. Women in childbearing years should be on a folic acid supplement when taking an anticonvulsant drug. In general, it appears that all men and women would benefit from increased folate intake. This can be accomplished through vitamin supplementation when there is compliance. However, if the food fortification for folate is increased in the future, then the issue of vitamin supplementation will have to be readdressed.

Exclusion reasons: review.

42. [Prim Care Update Ob Gyns.](http://www.ncbi.nlm.nih.gov/pubmed/?term=Folic+acid+and+preconceptional+care.+AND+Levine+NH) 2001 Mar;8(2):78-81.

# Folic acid and preconceptional care.

[Levine NH](http://www.ncbi.nlm.nih.gov/pubmed/?term=Levine%20NH%5BAuthor%5D&cauthor=true&cauthor_uid=11246033)1, [Lyon Daniel K](http://www.ncbi.nlm.nih.gov/pubmed/?term=Lyon%20Daniel%20K%5BAuthor%5D&cauthor=true&cauthor_uid=11246033), [Mulinare J](http://www.ncbi.nlm.nih.gov/pubmed/?term=Mulinare%20J%5BAuthor%5D&cauthor=true&cauthor_uid=11246033).

### [Author information](http://www.ncbi.nlm.nih.gov/pubmed/?term=Folic+acid+and+preconceptional+care.+AND+Levine+NH)

### Abstract

If all women capable of becoming pregnant consumed 400 µg (0.4 mg) of the B vitamin folic acid daily before conception and during the first trimester, the annual number of neural-tube birth defect (NTD)-affected pregnancies in this country could be reduced by 50% to 70%. Despite this important relationship, most women are not aware that folic acid prevents NTDs, and folic acid supplementation rates remain low. If folic acidconsumption is to succeed as a public health intervention on a societal scale, physicians who care for reproductive-age women must become informed about the folic acid-NTD prevention linkage. Next, they must be encouraged to counsel patients about the need to consume sufficient folic acid before conception. Studies reveal that few physicians have adequate knowledge of the appropriate timing and dosage of folic acid supplementation. Studies also show that women are more likely to get important folic acid information not from their physicians but from other sources. This article provides information about ways in which physicians can improve their folic acid knowledge and education practices.

Exclusion reasons: No autism data.

43. [J Nerv Ment Dis.](http://www.ncbi.nlm.nih.gov/pubmed/?term=Neurobiological+plausibility+of+prenatal+nutritional+deprivation+as+a+risk+factor+for+schizophrenia.) 1996 Feb;184(2):71-85.

# Neurobiological plausibility of prenatal nutritional deprivation as a risk factor for schizophrenia.

[Brown AS](http://www.ncbi.nlm.nih.gov/pubmed/?term=Brown%20AS%5BAuthor%5D&cauthor=true&cauthor_uid=8596115)1, [Susser ES](http://www.ncbi.nlm.nih.gov/pubmed/?term=Susser%20ES%5BAuthor%5D&cauthor=true&cauthor_uid=8596115), [Butler PD](http://www.ncbi.nlm.nih.gov/pubmed/?term=Butler%20PD%5BAuthor%5D&cauthor=true&cauthor_uid=8596115), [Richardson Andrews R](http://www.ncbi.nlm.nih.gov/pubmed/?term=Richardson%20Andrews%20R%5BAuthor%5D&cauthor=true&cauthor_uid=8596115), [Kaufmann CA](http://www.ncbi.nlm.nih.gov/pubmed/?term=Kaufmann%20CA%5BAuthor%5D&cauthor=true&cauthor_uid=8596115), [Gorman JM](http://www.ncbi.nlm.nih.gov/pubmed/?term=Gorman%20JM%5BAuthor%5D&cauthor=true&cauthor_uid=8596115).

### [Author information](http://www.ncbi.nlm.nih.gov/pubmed/?term=Neurobiological+plausibility+of+prenatal+nutritional+deprivation+as+a+risk+factor+for+schizophrenia.)

### Abstract

Emerging evidence indicates that schizophrenia may in some cases be a neurodevelopmental disorder, resulting in part from the effects ofprenatal exposures. Studies by our group have focused attention on the potential role of prenatal nutritional deficiency as a potential etiologicalfactor. Therefore, we sought to examine the biological plausibility of prenatal nutritional deprivation in the etiopathogenesis of schizophrenia. We conducted a review of the pertinent literature. Four lines of evidence support prenatal nutritional deficiencies as a plausible set of risk factors for schizophrenia: a) their effects are not incompatible with the epidemiology of schizophrenia; b) they have adverse effects on brain development; c) general malnutrition results in neuropathological anomalies of brain regions implicated in schizophrenia; and d) prenatalmalnutrition affects maternal systems critical to the developing fetal nervous system. There is sufficient evidence to warrant further studies ofprenatal nutritional deficits as risk factors for schizophrenia. A strategy for testing these hypotheses is outlined.

Exclusion reasons: Review.
